# A Blood‐Derived Factor Rescues ALS: Platelet Factor 4 Activates OPTN‐Dependent Autophagy to Clear SOD1 Aggregates Independently of PINK1

**DOI:** 10.1002/advs.76778

**Published:** 2026-07-23

**Authors:** Qingjian Xie, Yanbo Zhu, Wenhua Jiang, Haobo Xie, Yaojia Li, Mengjie Hu, Kezheng Li, Huan Yu, Yixuan Pan, Chaoyi Jiang, Xueqin Song, Dongsheng Fan, Binbin Deng

**Affiliations:** ^1^ Department of Rehabilitation Medicine First Affiliated Hospital of Wenzhou Medical University and Department of Neurology First Affiliated Hospital of Wenzhou Medical University Wenzhou China; ^2^ Department of Rehabilitation Medicine The Third Hospital of Hebei Medical University Shijiazhuang China; ^3^ Chinese PLA General Hospital Beijing China; ^4^ Department of Pediatrics Second Affiliated Hospital and Yuying Children's Hospital of Wenzhou Medical University Wenzhou China; ^5^ Department of Neurology and Neurological Laboratory of Hebei Province The Second Hospital of Hebei Medical University and Key Laboratory of Neurology (Hebei Medical University) Ministry of Education Shijiazhuang China; ^6^ Department of Neurology Peking University Third Hospital Beijing China

**Keywords:** amyotrophic lateral sclerosis, mitophagy, OPTN, PF4, proteostasis, SOD1

## Abstract

Peripheral factors that systemically regulate amyotrophic lateral sclerosis (ALS) have remained elusive‐until now. Here, by integrating population‐scale epidemiology with mechanistic dissection, we identify platelet factor 4 (PF4) as the central driver of a circulating neuroprotective axis that restores proteostasis and rescues ALS. In a prospective cohort of >500 000 UK Biobank participants, platelet indices were strongly associated with ALS risk, and serum PF4 levels were significantly reduced in ALS patients. Systemic administration of recombinant PF4 in hSOD1^G93A^ mice produced dramatic therapeutic effects: extended survival, preserved motor function, attenuated neuroinflammation, and reduced neuromuscular junction denervation. Remarkably, this efficacy appears pathology‐selective–robust in SOD1‐driven models but shows no observable effect in TDP‐43 or C9orf72 ALS models. Mechanistically, PF4 achieves what few molecules can: it engages the cell surface receptor LRP1 to activate the TBK1‐OPTN signaling axis, restoring impaired autophagic flux through a PINK1/Parkin‐independent pathway requiring ATG7, establishing a previously unrecognized peripheral platelet‐autophagy‐neuron axis that facilitates the co‐clearance of pathological SOD1 aggregates and damaged mitochondria. This study unveils PF4 as a first‐in‐class circulating autophagy regulator with therapeutic potential in ALS. Beyond identifying a candidate biomarker and drug lead, it reveals that systemic factors can directly engage central proteostatic machinery–opening a new frontier for ALS therapy.

## Graphical abstract

Systemic platelet factor 4 (PF4) is significantly depleted in amyotrophic lateral sclerosis (ALS). Peripheral PF4 replenishment restores central proteostasis by driving OPTN‐dependent, PINK1‐independent selective autophagy in motor neurons. This intervention effectively clears toxic SOD1 aggregates, blunts glial activation, and preserves neuromuscular junctions, ultimately extending survival and unveiling a novel circulating therapeutic axis for ALS.

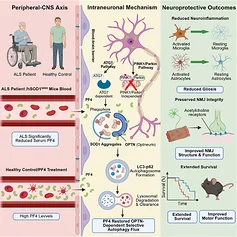

## 1 Introduction

Amyotrophic lateral sclerosis (ALS) is a relentless neurodegenerative disorder characterized by the selective loss of upper and lower motor neurons, culminating in paralysis and death typically within 2–4 years of diagnosis due to respiratory failure [1]. While the precise etiology of ALS remains incompletely understood, mounting evidence implicates protein misfolding, impaired proteostasis, mitochondrial dysfunction, compromised autophagy, and neuroinflammation as primary drivers of pathogenesis [2, 3, 4, 5]. Although mutations in genes such as *SOD1*, *TARDBP* (TDP‐43), and *C9orf72* in familial ALS have provided critical insights into disease mechanisms and therapeutic targets, effective treatment strategies remain scarce [6].

In recent years, attention has increasingly shifted toward potential systemic pathogenic contributors in ALS, including immune and hematological components [7, 8]. Among these, circulating platelets—beyond their classical role in hemostasis—have emerged as active regulators of neuroinflammation and neuronal survival through the release of trophic and inflammatory mediators [9, 10, 11, 12]. Altered platelet activity and the application of platelet‐derived signaling molecules have been reported in various neurodegenerative contexts, including Alzheimer's and Parkinson's diseases [13, 14]. However, whether platelet‐derived factors influence the onset or progression of ALS remains to be explored.

To address this gap, we first conducted a comprehensive population analysis using the UK Biobank (UKB) cohort, which offers extensive hematological and clinical follow‐up data from over 500 000 participants [15]. We observed that platelet‐related indices—including platelet count (PLT), plateletcrit (PCT), platelet distribution width (PDW), and mean platelet volume (MPV)—were significantly associated with ALS risk and survival. These findings prompted us to investigate whether specific platelet‐derived molecules might exert neuroprotective or disease‐modifying effects in ALS.

Among various platelet‐released factors—namely platelet factor 4 (PF4/CXCL4), brain‐derived neurotrophic factor (BDNF), and vascular endothelial growth factor (VEGF) [10]—we found that PF4 was among the most significantly downregulated factors in patients with ALS compared to age‐, sex‐, and comorbidity‐matched controls. PF4 is a CXC chemokine abundantly stored in platelet α‐granules [16] and is reported to regulate inflammation and angiogenesis, while participating in the activation, proliferation, and migration of various cell types [17]. Notably, Schroer et al. recently applied PF4 in murine aging models, revealing its multifaceted roles in attenuating neuroinflammation, enhancing synaptic plasticity markers, and modulating the immune system, thereby highlighting the significant translational value of PF4 in the nervous system [18]. Furthermore, in a survival study using SOD1^G86R^ mice, Gouel et al. found that intranasal administration of the <3 kDa fraction of human platelet lysate (HPPL)—in which PF4 is a major constituent—extended survival [8]. Nevertheless, the potential role and underlying mechanism of PF4 specifically in ALS remain largely uninvestigated.

We hypothesized that PF4 acts as a peripheral modulator of ALS pathogenesis by influencing neuronal proteostasis and mitochondrial quality control. Utilizing in vitro cell lines mimicking *SOD1*, *TARDBP*, and *C9orf72* mutations, in vivo transgenic hSOD1^G93A^ mice, and transcriptomic analysis, we systematically investigated the functional and mechanistic roles of PF4 in ALS. We found that PF4 selectively ameliorated SOD1‐associated pathology but was ineffective against TDP‐43 or C9orf72 poly(GR30/GA30)‐induced aggregation. Mechanistically, by engaging the LRP1 receptor, PF4 promotes autophagic flux and mitochondrial homeostasis via an OPTN‐dependent, but PINK1/Parkin‐independent, pathway, thereby enhancing the clearance of pathological protein aggregates and damaged mitochondria.

Our study unveils a previously unrecognized link between platelet factors and neuronal proteostasis, providing a potential therapeutic avenue for ALS that targets peripheral‐central communication.

## 2 Results

### 2.1 Platelet Indices Correlate with ALS Risk and Progression in the Uk Biobank Cohort

We first investigated the association between hematological parameters and ALS incidence within the UK Biobank (UKB) cohort (Figure 1A). Following multivariate adjustment, platelet‐related parameters—including platelet count (PLT), plateletcrit (PCT), and platelet distribution width (PDW)—exhibited significant correlations with both ALS incidence and disease progression (Figure 1B and Figure S1). Restricted cubic spline analyses revealed that elevated mean platelet volume (MPV) and PCT were inversely associated with ALS risk, whereas higher PDW was predictive of accelerated disease progression (Figure 1C). Correlation heatmaps and subgroup analyses further substantiated the robustness of these associations across populations stratified by age, sex, and body mass index (BMI) (Figure 1D–G). Collectively, these findings implicate platelet biology in the pathophysiology of ALS.

**FIGURE 1:**
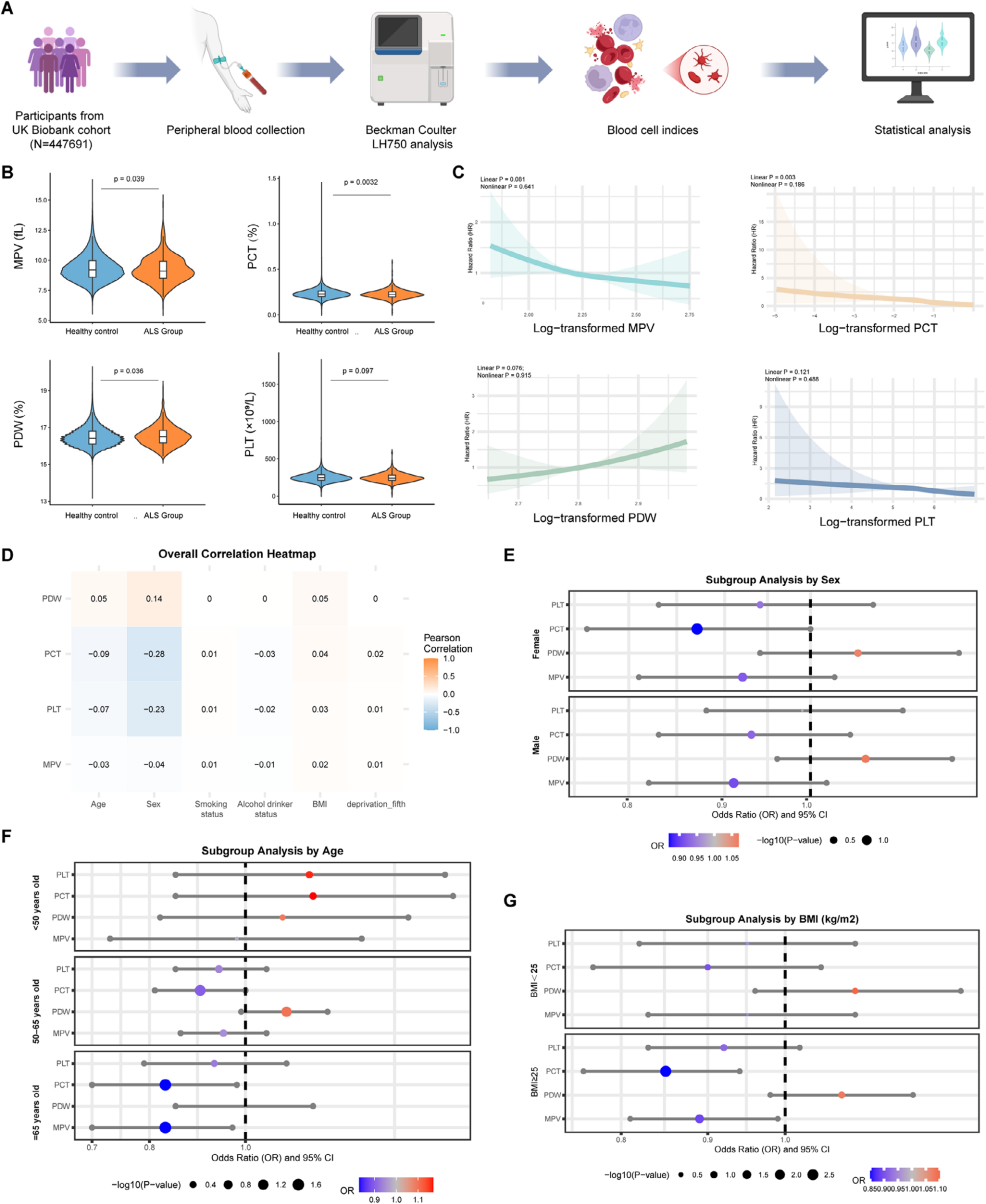
Association of platelet indices with ALS risk in the UK Biobank cohort. (A) Schematic workflow of the study design using the UK Biobank cohort (N = 447,691). (B) Violin plots comparing mean platelet volume (MPV), plateletcrit (PCT), platelet distribution width (PDW), and platelet count (PLT) between healthy controls and the ALS group. (C) Restricted cubic spline analyses showing the association between log‐transformed platelet indices and ALS risk (Hazard Ratio, HR). Shaded areas represent 95% confidence intervals (CI). (D) Heatmap of Pearson correlation coefficients between platelet indices and covariates including age, sex, smoking/alcohol status, BMI, and deprivation index. (E–G) Subgroup analyses stratified by sex (E), age (F), and BMI (G). Circles represent the Odds Ratio (OR) and horizontal lines indicate the 95% CI. Color gradients reflect the statistical significance level (‐log10 *p*‐value). Statistical comparisons in (B) were performed using the Student's t‐test. Exact *p* values are indicated.

### 2.2 ALS Drives a Specific Depletion of Serum PF4 Independent of Physiological Aging

The pivotal role of platelet factors in neurodegenerative diseases has been substantiated by multiple studies. To comprehensively investigate potential therapeutic targets and eliminate selection bias, we first conducted a multiplex profiling of 10 representative platelet‐associated and neurotrophic factors across strictly matched cohorts of healthy controls (HC), ALS, Alzheimer's disease (AD), and Parkinson's disease (PD) patients (*n* = 60 per group) (Figure S2A). Strikingly, this broad screening revealed that the significant depletion of PF4 was highly specific to the ALS cohort, whereas its circulating levels remained relatively stable in both AD and PD patients (Figure S2B–D).

To rigorously validate this ALS‐specific PF4 deficit and resolve the confounding effects of sample size and physiological aging, we subsequently expanded our primary cohort to 100 ALS patients and 100 age‐matched HCs, focusing on PF4 alongside BDNF and VEGF —which collectively reflect vascular, neurotrophic, and stress‐related pathways [19, 20, 21]. Consistent with our initial multiplex data, VEGF remained stable, whereas both BDNF and PF4 exhibited significant reductions in the expanded ALS group (*p* < 0.05; Figure 2A). While the BDNF reduction aligns with general neurotrophic deficits commonly reported across various neurodegenerative contexts [22, 23, 24], the profound and disease‐specific downregulation of PF4 warrants unique attention.

**FIGURE 2:**
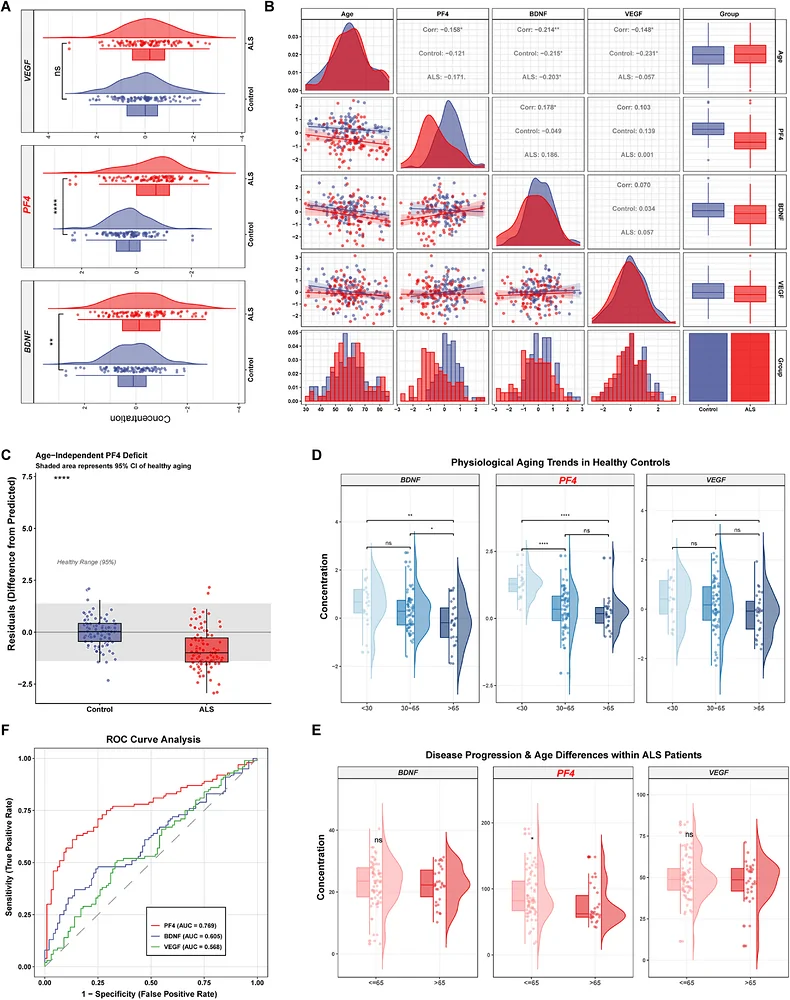
Comparative analysis of serum PF4, BDNF, and VEGF levels in the expanded cohort of healthy controls and ALS patients. (A) Raincloud plots showing the standardized serum concentrations of VEGF, PF4, and BDNF in age‐matched healthy controls and ALS patients. Importantly, to eliminate inter‐assay variability (batch effects) across two independent ELISA batches, raw absolute concentrations were subjected to Z‐score standardization prior to pooled analysis. The Y‐axes thus represent these dimensionless standardized values. (B) Multivariable correlation matrix of age and biomarker levels. Diagonal panels display density distributions. Upper triangle panels show Pearson's correlation coefficients (r). Lower triangle panels display scatter plots with linear regression lines and 95% confidence intervals (shaded areas) for healthy controls (red) and ALS patients (blue). (C) Age‐adjusted residual analysis for PF4 (Age‐Independent PF4 Deficit). Residuals represent the difference between observed levels and values predicted by the healthy aging model. The grey shaded area indicates the 95% confidence interval (CI) of the healthy reference range. (D) Analysis of standardized biomarker levels in healthy controls stratified by physiological aging stages (<30, 30–65, and >65 years). (E) Biomarker levels within the ALS cohort stratified by age at sampling (≤65 versus >65 years). (F) Receiver operating characteristic (ROC) curves evaluating the diagnostic performance of PF4 (red, AUC = 0.769), BDNF (blue, AUC = 0.605), and VEGF (green, AUC = 0.568) in distinguishing ALS patients from healthy controls. Data are presented as individual data points with box‐and‐whisker plots or violin plots from independent human serum samples (n = 100 per group). Statistical significance was determined using an unpaired Student's t‐test for comparing standardized concentrations (Panel A) and age‐stratified subgroups (Panel E). Specifically, for the age‐adjusted residual analysis (Panel C), an unpaired Student's t‐test was utilized to compare the distribution of residuals in the ALS group against those of the healthy controls. One‐way ANOVA followed by Tukey's post hoc test was applied for multi‐group comparisons (Panel D). *p < 0.05, *p* < 0.01, ***p < 0.001, ****p < 0.0001; ns = not significant.

To investigate the potential of platelet‐derived factors as therapeutic targets for ALS, we quantified plasma levels of PF4, BDNF, and VEGF in an age‐matched cohort of ALS patients and healthy controls (HC). Unlike VEGF, which remained stable, both BDNF and PF4 exhibited significant reductions in the ALS group (*p* < 0.05; Figure 2A). While BDNF reduction aligns with general neurotrophic deficits, the profound downregulation of PF4 warrants specific attention.

Given that PF4 levels naturally fluctuate with age, we performed a multivariable correlation analysis (Figure 2B). As expected, HCs showed a physiological decline in PF4 with age (r = −0.316, *p* < 0.05). Crucially, while the ALS group maintained a similar age‐related trend, their PF4 levels were consistently suppressed relative to controls across the entire age spectrum (r = −0.593). To strictly quantify this disease‐specific loss, we calculated age‐adjusted residuals (Figure 2C). Although ALS residuals did not completely fall outside the healthy physiological range, they exhibited a robust negative shift (*p* < 0.0001), demonstrating a systemic “PF4 gap” that cannot be attributed to age alone.

We further dissected this age‐dependent dynamic through stratified subgroup analyses. In the extended HC cohort, PF4 levels exhibited a stepwise physiological decline from young adulthood (<30y) to senescence (>65y) (Figure 2D). Interestingly, this age‐dependent loss was also preserved within the ALS cohort, where late‐onset patients (>65y) exhibited significantly lower PF4 levels compared to early‐onset patients (≤65y) (Figure 2E), suggesting a “double hit” of disease pathology and biological aging.

Finally, regarding diagnostic utility, ROC analysis indicated moderate discriminatory power for PF4 (AUC = 0.614), comparable to BDNF (Figure 2F). Although its standalone diagnostic sensitivity is limited due to physiological overlap, the identification of a specific, age‐independent PF4 deficit supports its value as a mechanistic biomarker linking peripheral hematological dysfunction to neurodegeneration.

### 2.3 PF4 Selectively Attenuates SOD1 Aggregation and Enhances Cell Viability

To examine whether PF4 exerts protective effects against ALS‐associated proteinopathies, we treated cells expressing EGFP‐SOD1^G93A^, EGFP‐TDP‐25, or EGFP–poly(GR/GA30) with recombinant mouse PF4 (Figure 3A,I). PF4 treatment markedly reduced SOD1 protein levels and aggregate formation, while concurrently rescuing cell viability (Figure 3B–H). Conversely, PF4 failed to alleviate pathological protein aggregation or mitigate cytotoxicity in either the TDP‐25 (Figure 3J–N) or poly(GR/GA30) models (Figure 3O–T). These data demonstrate that PF4 selectively ameliorates SOD1‐associated proteotoxicity.

**FIGURE 3:**
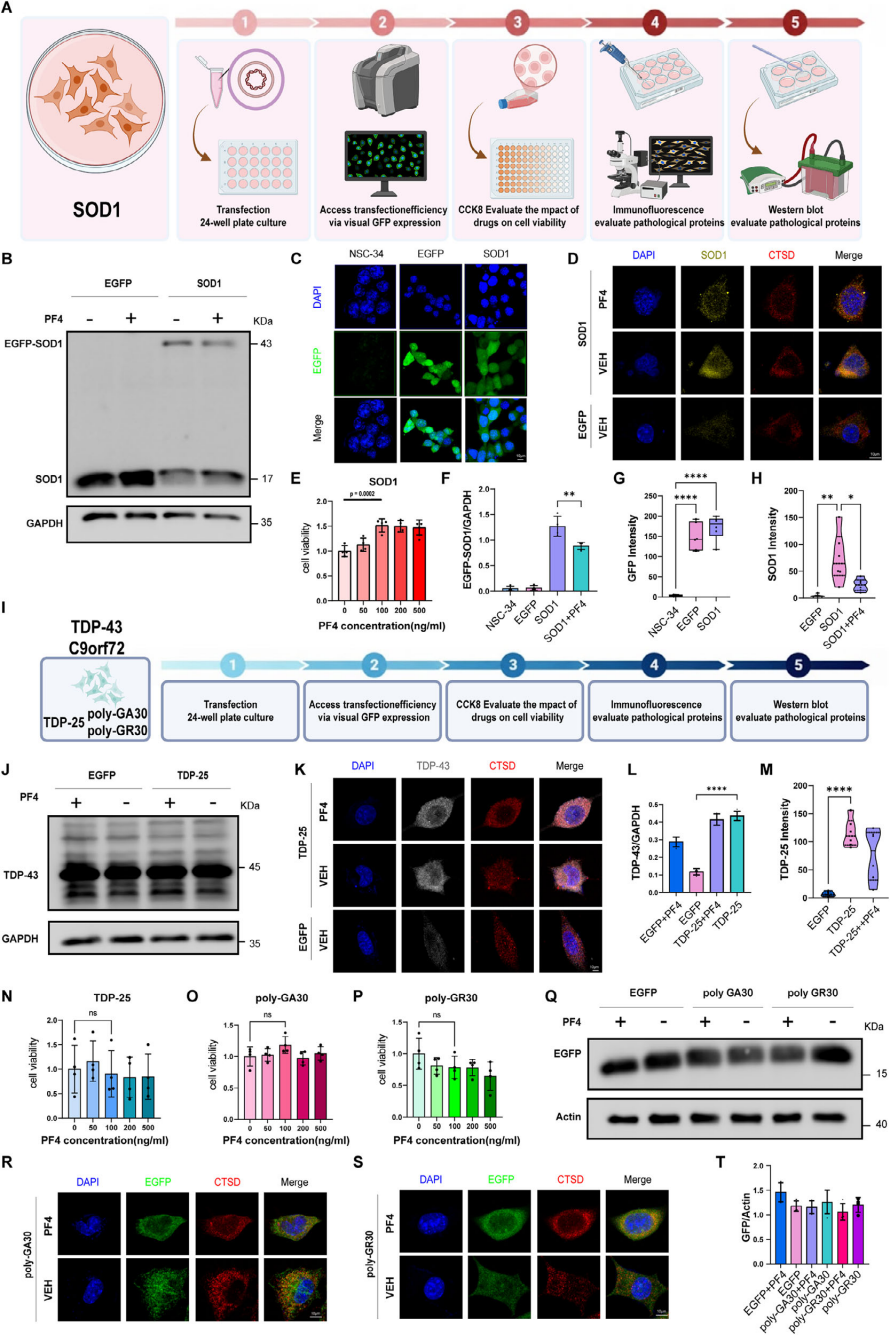
Selective effects of PF4 on SOD1, TDP‐43, and C9orf72 pathology in vitro. (A) Schematic workflow for assessing PF4 effects in cells expressing mutant SOD1. (B–H) Analysis of SOD1 pathology. (B) Representative western blots of EGFP‐SOD1 protein levels. (C,D) Immunofluorescence images evaluating visual GFP expression (C) and colocalization of SOD1 with CTSD (D) across different experimental conditions. (E) Cell viability assessed by CCK‐8 assay at varying PF4 concentrations. (F) Quantification of EGFP‐SOD1 protein levels normalized to GAPDH. (G, H) Quantification of GFP intensity (G) and SOD1 intensity (H). (I) Schematic workflow for assessing PF4 effects in cells expressing TDP‐25 and C9orf72 dipeptide repeats (poly‐GA30 and poly‐GR30). (J–N) Analysis of TDP‐43 (TDP‐25) pathology. (J) Representative western blots of TDP‐43 and EGFP protein levels in TDP‐25 models. (K) Immunofluorescence images showing TDP‐43 and CTSD distribution. (L) Quantification of TDP‐43 protein levels normalized to GAPDH. (M) Quantification of TDP‐25 intensity. (N) Cell viability of the TDP‐25 model assessed by CCK‐8 assay at varying PF4 concentrations. (O–T) Analysis of C9orf72 pathology. (O,P) Cell viability assessed by CCK‐8 assay for poly‐GA30 (O) and poly‐GR30 (P) models. (Q) Representative western blots of EGFP levels in poly‐GA30 and poly‐GR30 groups. (R,S) Immunofluorescence images of EGFP and CTSD in poly‐GA30 (R) and poly‐GR30 (S) models. (T) Quantification of GFP protein levels normalized to Actin. Data are presented as mean ± SD from independent biological replicates (*n* = 4 for western blot; *n* = 3 for CCK‐8 assays; *n* = 6 for immunofluorescence quantifications). Statistical significance was determined using one‐way ANOVA followed by Tukey's post hoc test. *p < 0.05, *p* < 0.01, ***p < 0.001, ****p < 0.0001; ns = not significant.

### 2.4 PF4 Delays Disease Progression and Reduces Pathological Protein Burden in hSOD1^G93A^ Mice

Before evaluating therapeutic efficacy, we first investigated the systemic dynamics and central penetrance of PF4 in vivo. Similar to the clinical ALS cohort, hSOD1^G93A^ mice exhibited a progressive decline in plasma PF4 levels as the disease advanced (Figure S3A). Crucially, biodistribution assays following extensive transcardial perfusion confirmed that peripherally administered PF4 (via both i.v. and i.p. routes) successfully crossed the intact blood‐brain barrier (BBB), accumulating significantly in both the brain and spinal cord parenchyma (Figure S3B–D).

Having established target engagement in the CNS, we evaluated the dose‐dependent clearance of mutant SOD1 in the spinal cord to optimize the in vivo dosage (Figure S4A,B). Subsequently, we administered recombinant PF4 to hSOD1^G93A^ mice across two distinct clinical intervention windows: presymptomatic (P60) and post‐onset (P90). While PF4 treatment initiated at P60 did not significantly alter disease onset (Figure 4F), it substantially extended both overall survival and disease duration (Figure 4G–H). PF4‐treated mice exhibited a marked delay in weight loss (Figure 4C–E) and demonstrated superior performance in neurological scoring, rotarod, grip strength, and wire hang tests, indicating effective preservation of motor function (Figure 4I–M). Remarkably, this robust neuroprotective and disease‐modifying efficacy was partially preserved even when intervention was delayed until the onset of overt motor symptoms at P90 (Figure S4E–J), although early presymptomatic intervention (P60) yielded superior efficiency in clearing mutant SOD1 aggregates (Figure S4C,D).

**FIGURE 4:**
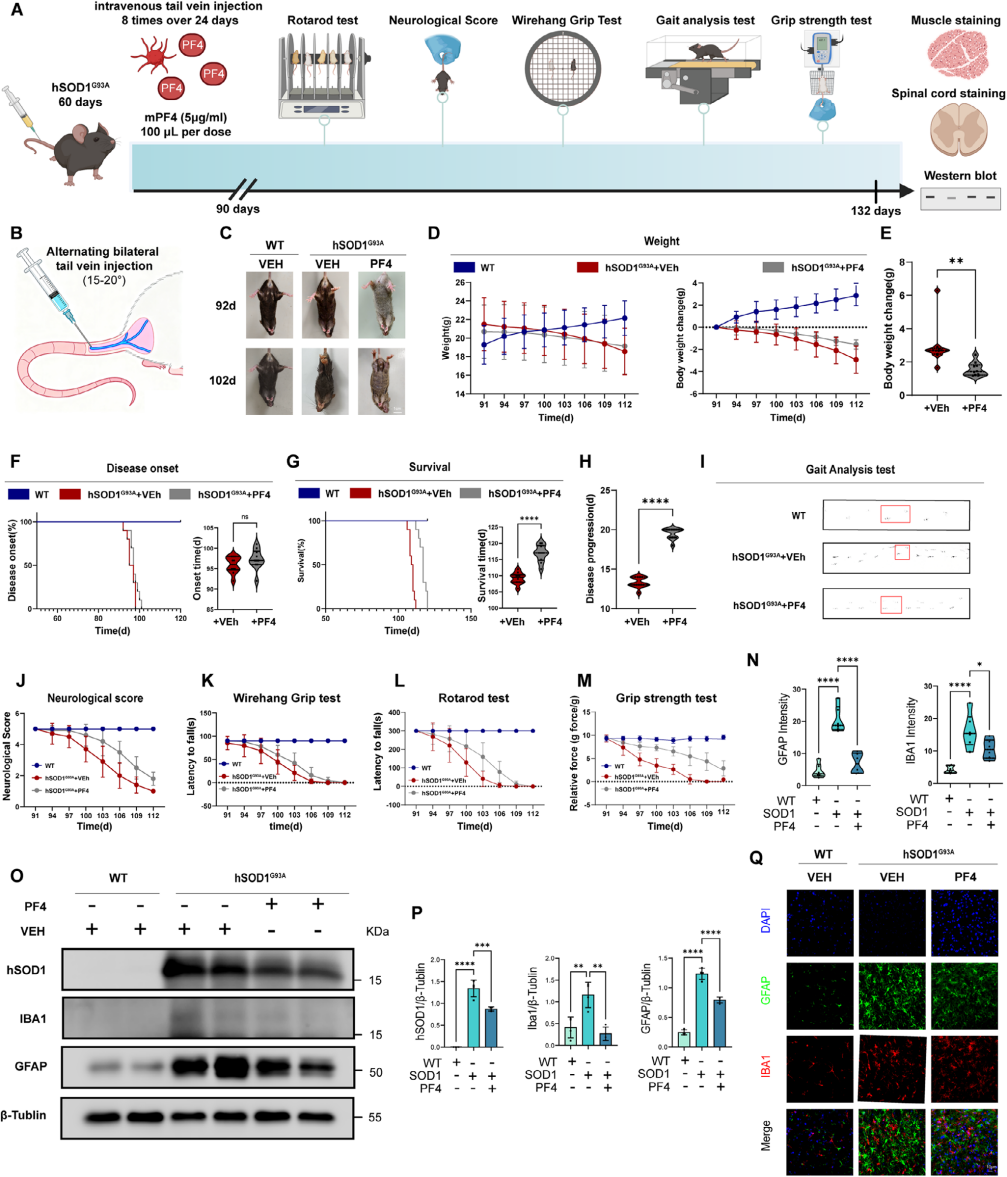
Effects of PF4 treatment on survival, motor function, and neuroinflammation in hSOD1^G93A^ mice. (A,B) Experimental design (A) and tail vein injection strategy (B). hSOD1^G93A^ mice received recombinant PF4 or vehicle treatment starting at postnatal day 60. (C–E) Analysis of body weight. Representative images of mice at 92 and 102 days (C). Scale bars = 1 cm. Longitudinal body weight measurements (D) and body weight change (E). (F–H) Disease course analysis. Disease onset curves and absolute onset times (F), Kaplan‐Meier survival curves and absolute survival times (G), and disease progression duration (H). (I–M) Assessment of motor function. Representative gait footprints (I). Longitudinal analysis of neurological score (J), wire hang test (K), rotarod test (L), and grip strength (M). (O,P) Western blot analysis of spinal cord lysates. Representative blots (O) and quantification (P) of mutant hSOD1, Iba1, and GFAP levels normalized to β‐tubulin. (N,Q) Immunofluorescence analysis of the spinal cord ventral horn. Representative images (N) showing DAPI (blue), GFAP (green), and Iba1 (red) staining. Quantification of fluorescence intensity (Q). Scale bars = 10 µm. Data are presented as mean ± SEM (or visualized as violin plots to show individual data distribution) from independent biological replicates (*n* = 4 for western blot assays; *n* = 6 for immunofluorescence quantifications; *n* = 10 mice per group for in vivo behavioral and survival analyses). Statistical significance was determined using the Log‐rank (Mantel‐Cox) test for Kaplan‐Meier survival and onset curves (left panels in F and G), and the one‐way ANOVA followed by Tukey's post hoc test for multi‐group comparisons (O,Q). Notably, for the two‐group comparisons of data presented in violin plots (E, H, and right panels in F and G), the non‐parametric Mann‐Whitney U test was applied to account for potential non‐normal distributions. *p < 0.05, *p* < 0.01, ***p < 0.001, ****p < 0.0001; ns = not significant.

To dissect the underlying mechanisms, we assessed the pathological burden within the spinal cord. Quantitative Western blotting revealed that PF4 treatment significantly reduced the levels of mutant hSOD1 protein (Figure 4N,Q). Furthermore, quantification of neuroinflammatory markers confirmed that PF4 markedly suppressed glial hyperactivation, evidenced by the substantial reduction in protein expression and fluorescence intensity of Iba1 (microglia) and GFAP (astrocytes) in the spinal cord ventral horns (Figure 4O,P). This potent anti‐inflammatory effect was further corroborated by absolute quantitative ELISA profiling, which demonstrated that systemic PF4 profoundly blunted the expression cascade of core pro‐inflammatory mediators—including TNF‐α, C1q, CD11b, IL‐1β, and NF‐κB—in the spinal cord microenvironment (Figure S3E). These data indicate that PF4 effectively mitigates the core molecular pathologies driving ALS progression in vivo.

### 2.5 PF4 Exerts Broad Protective Effects on Central Motor Neurons and Peripheral Neuromuscular Integrity

Morphological assessment revealed that PF4 treatment exerted broad protective effects extending from the spinal cord to peripheral effector organs. In the spinal cord ventral horns, both immunofluorescence and immunohistochemical analyses of NeuN confirmed that PF4 preserved motor neuron numbers and mitigated neuronal atrophy (Figure 5B–F). Nissl staining further demonstrated the preservation of Nissl bodies and a significant reduction in chromatolysis in the PF4‐treated group (Figure 5G,H). This was accompanied by a marked amelioration of neuroinflammation, characterized by significantly reduced density and activation of Iba1+ microglia and GFAP+ astrocytes (Figure 5I–L).

**FIGURE 5:**
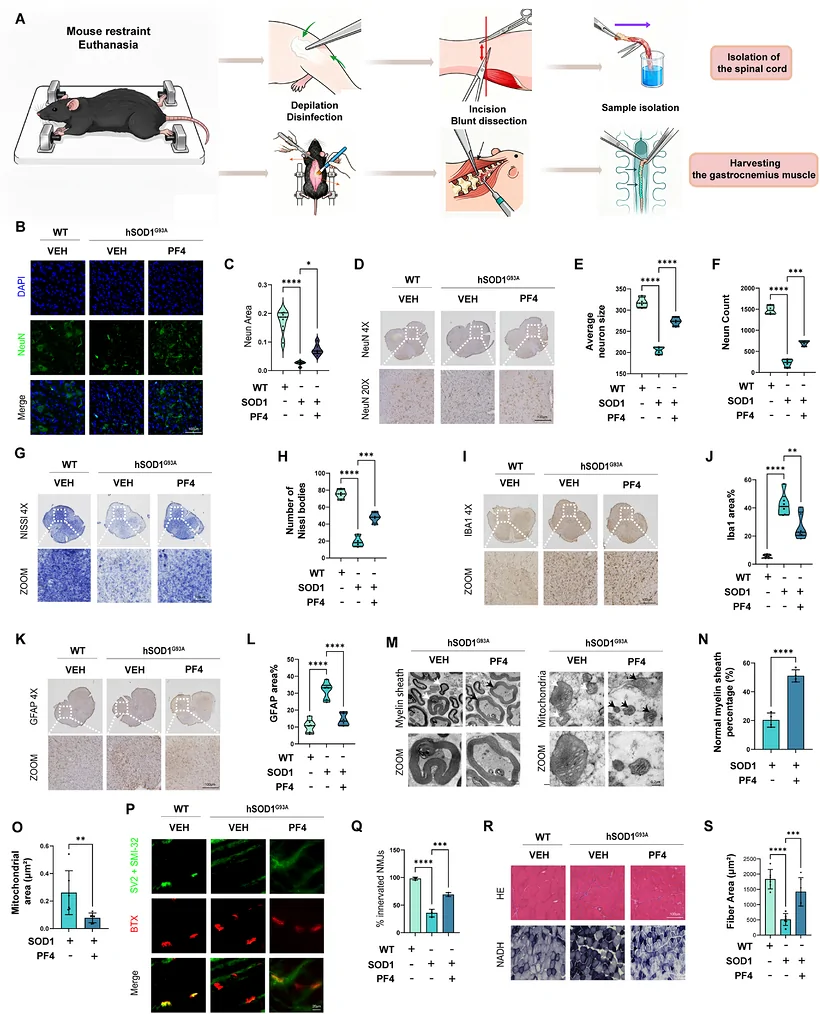
PF4 exerts broad neuroprotective effects extending from the central spinal cord to peripheral effector organs in hSOD1^G93A^ mice. (A) Schematic illustration of the experimental workflow, including mouse restraint, euthanasia, and isolation of the spinal cord and gastrocnemius muscle. (B,C) Representative immunofluorescence images of NeuN (green) in the lumbar spinal cord ventral horns (B) and quantification of NeuN area (C). Scale bar = 100 µm. (D–F) Representative immunohistochemical (IHC) staining of NeuN (top: 4X; bottom: 20X zoom) (D), along with quantitative analyses of average neuron size (E) and NeuN cell counts (F). Scale bars = 100 µm. (G,H) Representative Nissl staining (G) and quantification of surviving Nissl bodies (H). Scale bars = 100 µm. (I–L) Representative IHC images and quantification of Iba1 microglia (I,J) and GFAP astrocytes (K,L) in the ventral horns. Scale bars = 100 µm. (M–O) Transmission electron microscopy (TEM) analysis of the sciatic nerve. (M) Representative TEM micrographs showing myelin sheaths (left, scale bar = 2 µm) and intra‐axonal mitochondria (right, scale bar = 0.2 µm). White boxes indicate the regions of interest extracted for the high‐magnification bottom panels. (N) Quantification of normal myelin sheaths. (O) Quantitative measurement of individual mitochondrial area. (P,Q) Representative immunofluorescence images of neuromuscular junctions (NMJs) (P). Pre‐synaptic terminals are marked by SV2 and SMI‐32 (green), and post‐synaptic receptors by α‐BTX (red). Scale bar = 20 µm. (Q) Quantification of innervated NMJs. (R,S) Representative H&E and NADH staining of the gastrocnemius muscle (R) and quantification of muscle fiber area (S). Scale bars = 100 µm. Data are presented as mean ± SEM (or visualized as violin plots) from independent biological replicates (*n* = 6 for spinal cord and muscle histological/immunofluorescence quantifications; *n* = 3 for NMJ colocalization analysis; *n* = 6 for TEM analysis). Statistical significance was determined using one‐way ANOVA followed by Tukey's post hoc test for multi‐group analyses (Panels C, E, F, H, J, L, Q, S), and an unpaired Student's t‐test for normally distributed two‐group analyses (Panel N). Specifically, the non‐parametric Mann‐Whitney U test was applied for single‐organelle quantification of mitochondrial area (Panel O). *p < 0.05, *p* < 0.01, ***p < 0.001, ****p < 0.0001.

This neuroprotection extended to the peripheral nervous system. Transmission electron microscopy (TEM) analysis of sciatic nerves revealed severe ultrastructural damage in the vehicle controls, characterized by loose and in folded myelin lamellae, as well as profound mitochondrial swelling with disrupted cristae. Notably, PF4 treatment effectively prevented these morphological aberrations (Figure 5M). These ultrastructural observations were strongly supported by quantitative analyses, which confirmed that PF4 significantly rescued the percentage of normal myelin sheaths (Figure 5N) and robustly reduced pathological mitochondrial enlargement (Figure 5O). At the synaptic level, PF4 significantly alleviated neuromuscular junction (NMJ) denervation, evidenced by the preserved colocalization of pre‐synaptic terminals (marked by SV2 and SMI‐32) with post‐synaptic receptors (marked by α‐BTX) (Figure 5P,Q). Concomitantly, downstream muscle pathology was improved; H&E and NADH staining of the gastrocnemius muscle confirmed that PF4 reduced myofiber atrophy and maintained metabolic integrity (Figure 5R,S).

### 2.6 PF4 Induces Global Transcriptional Reprogramming and Reverses Mitochondrial and Autophagic Stress Signatures in SOD1 Model Cells

To ground our investigation in established pathology, we first analyzed a published transcriptomic dataset of SOD1‐ALS iPSC‐derived motor neurons (GSE158264) [25](Figure S5A). Both GO enrichment and GSEA analyses revealed that differentially expressed genes (DEGs) were significantly enriched in biological processes governing autophagy and mitochondrial function, specifically “autophagosome organization” and “mitochondrial gene expression” (Figures S5D–F and S6A,B). Notably, core genes such as *PINK1*, *PRKN*, and *SQSTM1* exhibited distinct expression profiles (Figure S5B,C). This a priori evidence from an independent dataset establishes dysregulated autophagy and mitochondrial homeostasis as core transcriptomic signatures of SOD1‐ALS.

To rigorously define whether PF4 reverses these pathological mechanisms, we performed RNA sequencing on SOD1 model cells treated with PF4 or vehicle (VEH). Principal component analysis (PCA) revealed that PF4 induced a profound and consistent global shift in gene expression, with the treated (SOD1_PF4) and control (SOD1_VEH) groups forming two distinct, non‐overlapping clusters (Figure 6A). This clear separation was further substantiated by the sample correlation matrix: while all samples maintained high correlation, intra‐group consistency (R^2^ ≈ 0.98–1.0) consistently exceeded inter‐group correlation (R^2^ ≈ 0.92–0.97), indicating that PF4 elicits specific therapeutic transcriptional reprogramming (Figure 6B). Differential expression analysis identified thousands of significantly altered transcripts (Figure 6C), while Venn diagrams showed that both groups shared the vast majority of the expressed gene pool, suggesting that PF4 modulates the magnitude of gene expression rather than functioning through binary on/off switching (Figure 6D).

**FIGURE 6:**
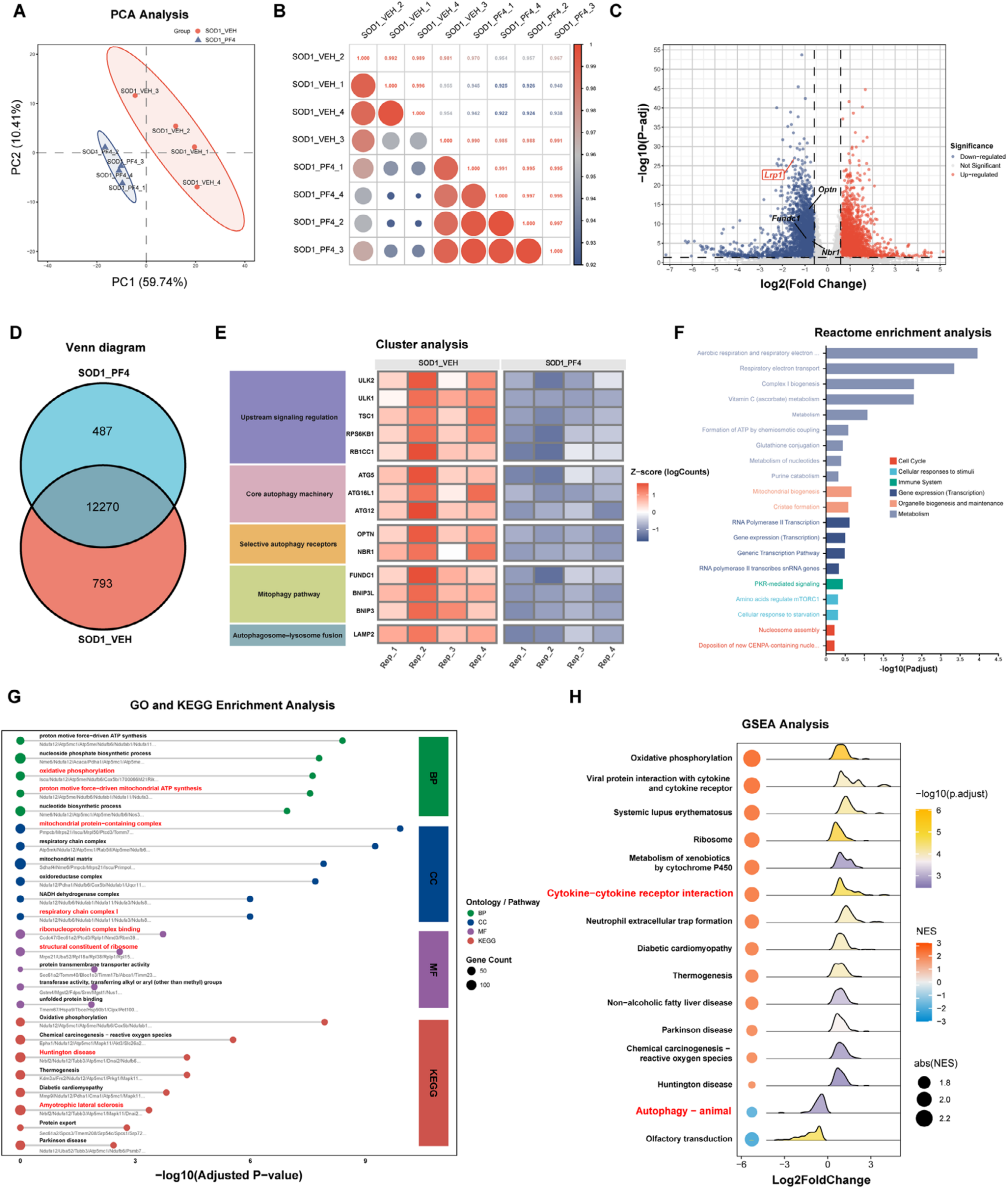
Transcriptomic analysis of SOD1 model cells following PF4 treatment. (A,B) Global gene expression profiling. Principal component analysis (PCA) **(A)** and Pearson correlation matrix (B) of vehicle‐treated (SOD1_VEH) and PF4‐treated (SOD1_PF4) cells. (C,D) Analysis of differentially expressed genes (DEGs). Volcano plot (C) displaying DEGs; red and blue points represent significantly upregulated and downregulated genes, respectively. Vertical and horizontal dashed lines indicate the log2(Fold Change) and adjusted *p*‐value thresholds. Venn diagram (D) showing the overlap of expressed genes between groups. (E) Hierarchical clustering heatmap of selected genes related to upstream signaling, core autophagy machinery, selective autophagy receptors, mitophagy, and autophagosome‐lysosome fusion. The color scale represents row‐scaled Z‐scores (red = high expression; blue = low expression). (F–H) Pathway enrichment analyses. Reactome pathway analysis (F) based on DEGs. Dot plot of Gene Ontology (GO) and KEGG pathway enrichment analysis (G). Gene Set Enrichment Analysis (GSEA) (H) ridge plots showing normalized enrichment scores (NES) for key pathways including oxidative phosphorylation and neurodegenerative disease‐related signatures. Color gradients and dot sizes indicate statistical significance (‐log10 adjusted *p*‐value) and gene counts. Transcriptomic data were derived from independent biological replicates (*n* = 4 per group). For differential gene expression analysis (C,D), statistical significance was evaluated using the Wald test (via DESeq2 pipeline) followed by the Benjamini‐Hochberg procedure for multiple testing correction. DEGs were strictly defined by an adjusted *p*‐value (P‐adj) < 0.05 and appropriate |log2(Fold Change)| thresholds. For functional enrichment analyses including Reactome, GO, KEGG, and GSEA (F–H), statistical significance was determined using hypergeometric tests or permutation‐based statistical methods, with significance rigorously defined by adjusted P‐values as indicated by the respective color scales.

Building on this context, we adopted a hypothesis‐driven strategy focusing on core autophagy, mitophagy, and lysosomal genes. Applying strict statistical thresholds (|log2FC| > 0.585, FDR < 0.05), we identified 14 pivotal genes (Figure 6E). Heatmap clustering revealed a highly consistent pattern: in the Control_VEH group, key genes—including *ULK1/2*, *TSC1*, *OPTN*, and *BNIP3*—were predominantly upregulated (red), indicative of high basal activation of stress response pathways in the SOD1 model. In sharp contrast, PF4 treatment induced a concerted transcriptional downregulation (blue) of this gene set. This transcriptional downregulation likely reflects the alleviation of feedback loop signaling, as the restoration of functional protein degradation reduces the demand for compensatory gene expression.

To place this phenomenon in a broader context, we subsequently performed unbiased enrichment analysis. Reactome, GO, and KEGG pathway analyses consistently showed that DEGs were heavily enriched in pathways related to “mitochondrial protein complex,” “NADH dehydrogenase complex,” and “oxidative phosphorylation” (Figure 6F,G), pointing to a comprehensive recovery of mitochondrial function. Crucially, Gene Set Enrichment Analysis (GSEA) not only confirmed the upregulation of oxidative phosphorylation but also unveiled a defining feature: the massive, systemic upregulation of the “Ribosome” pathway (Figure 6H).

Collectively, the transcriptomic data reveal a complex and precise cellular response to PF4: a targeted transcriptional downregulation of the autophagic stress axis (Figure 6E) accompanied by a systemic upregulation of the translation machinery and mitochondrial metabolism (Figure 6H). This suggests that the primary mechanism of PF4 involves the alleviation of cellular stress and the enhancement of metabolic and synthetic capacity via post‐transcriptional regulation.

### 2.7 PF4 Enhances Lysosome‐Dependent Autophagic Flux

To validate the alterations in autophagic flux suggested by our transcriptomic data, we assessed key autophagy markers. Immunoblotting and immunofluorescence analyses revealed elevated LC3‐II/I ratios and p62 levels in SOD1‐expressing cells (Figure 7B,C). Notably, the spontaneous aggregation patterns of LC3 and p62 were comparable to those observed in control cells treated with chloroquine (CQ), indicative of a blockade in basal autophagic flux within the SOD1 model [26, 27] (Figure 7F–H and Figure S7).

**FIGURE 7:**
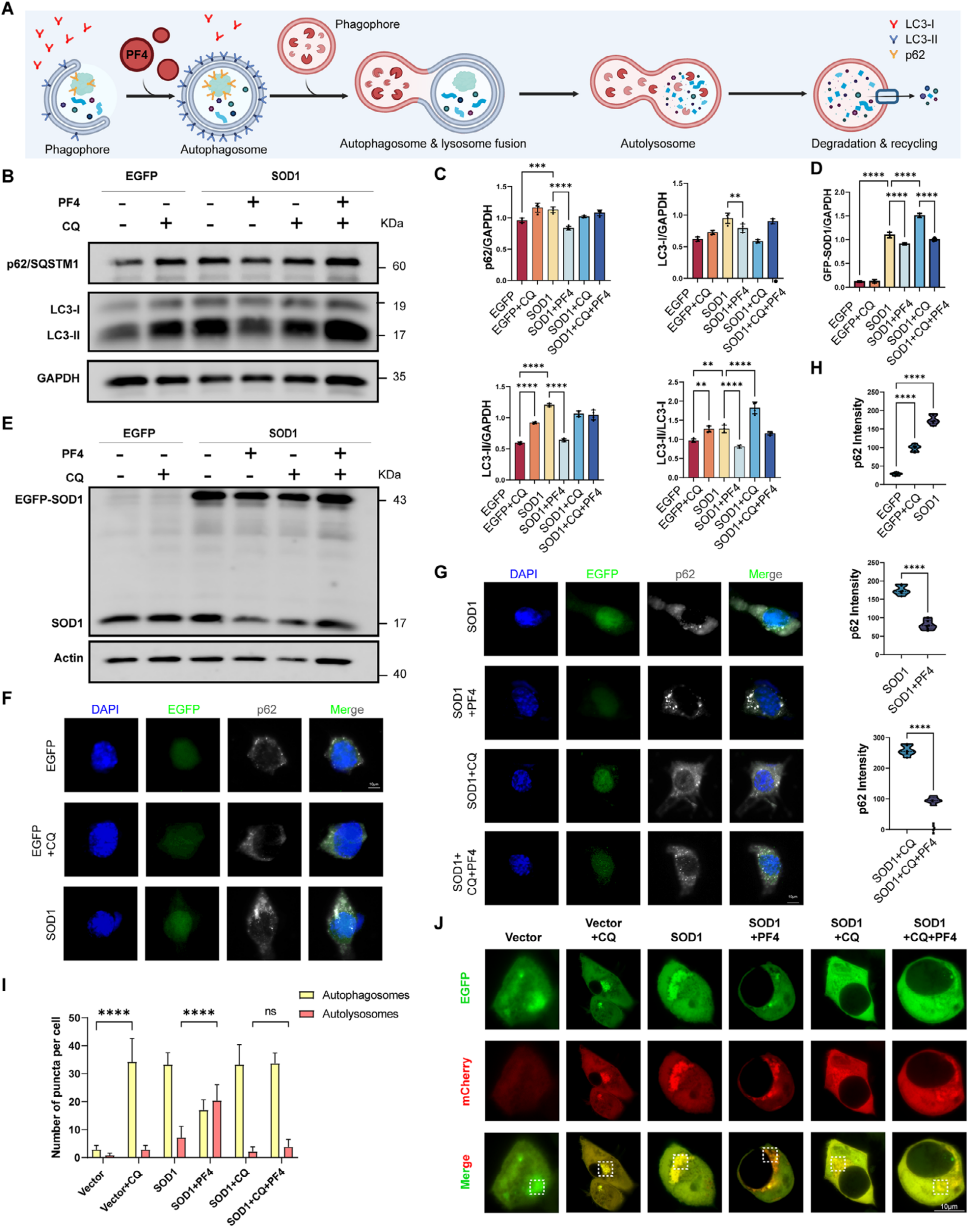
PF4 restores autophagic flux and promotes lysosome‐dependent degradation of mutant SOD1. (A) Schematic illustration of the autophagic pathway and the proposed mechanism of PF4‐mediated clearance. (B,C) Western blot analysis of autophagic flux markers. Representative blots (B) and corresponding densitometric quantification (C) of p62/SQSTM1 and LC3 (including LC3‐I, LC3‐II, and LC3‐II/LC3‐I ratio) levels in EGFP and SOD1 cells treated with or without PF4 and the lysosomal inhibitor chloroquine (CQ). (D,E) Evaluation of mutant SOD1 protein clearance. Representative Western blots (E) of EGFP‐SOD1 and endogenous SOD1 levels, with the corresponding quantification of EGFP‐SOD1 protein levels (D). (F–H) Immunofluorescence analysis of p62 accumulation and clearance. Representative confocal images showing p62 puncta (gray) in basal/control groups (F) and PF4/CQ treatment groups (G). Quantification of mean p62 fluorescence intensity across the indicated groups is shown in (H). (I,J) Direct measurement of dynamic autophagic flux using the tandem mCherry‐EGFP‐LC3 reporter. Representative confocal images (J) displaying the distribution of EGFP (green) and mCherry (red) signals across the six experimental groups. Quantification (I) of the average number of autophagosomes (yellow puncta in the merged images) and autolysosomes (red‐only puncta) per cell. Data are presented as mean ± SEM. Statistical significance was determined using one‐way ANOVA followed by Tukey's post hoc test for single‐variable comparisons (C, D, H), and two‐way ANOVA followed by Sidak's multiple comparisons test for the tandem reporter puncta quantification (I). For Western blot assays, *n* = 4 independent biological replicates per group. For immunofluorescence quantification, *n* = 20–30 cells per group from 6 independent experiments. *p < 0.05, *p* < 0.01, *p < 0.001, ****p < 0.0001; ns, not significant. Scale bars = 10 µm.

PF4 treatment significantly reduced p62 levels and the LC3‐II/I ratio, signaling the restoration of autophagic flux (Figure 7B,C,F–H). Concurrently, levels of pathological SOD1 protein were diminished. However, co‐treatment with the lysosomal inhibitor CQ completely abolished this effect, resulting in the re‐accumulation of SOD1 protein (Figure 7D,E).

To definitively confirm the dynamic restoration of autophagic flux, we utilized a tandem mCherry‐EGFP‐LC3 reporter in cells expressing a non‐fluorescent SOD1 construct to avoid spectral overlap. This assay unequivocally demonstrated that PF4 promoted the robust maturation of autophagosomes (yellow puncta) into autolysosomes (red‐only puncta), a degradative process that was completely blocked by CQ (Figure 7I,J). Thus, PF4 promotes autophagic turnover via a mechanism strictly dependent on lysosomal degradation.

### 2.8 PF4 Action Is Dependent on ATG7

To investigate whether PF4 modulates the upstream initiation of autophagy, we focused on ATG7, the critical E1‐like enzyme mediating LC3 conjugation and autophagosome biogenesis [28, 29] (Figure 8A). After validating siRNA knockdown efficiency via Western blotting and immunofluorescence (Figure 8B–E), we observed that PF4 treatment itself did not alter basal ATG7 protein levels (Figure 8G,I). However, under conditions where autophagic flux was compromised by ATG7 silencing, PF4 supplementation failed to ameliorate the accumulation of p62 or the elevated LC3‐II/LC3‐I ratio (Figure 8G,I).

**FIGURE 8:**
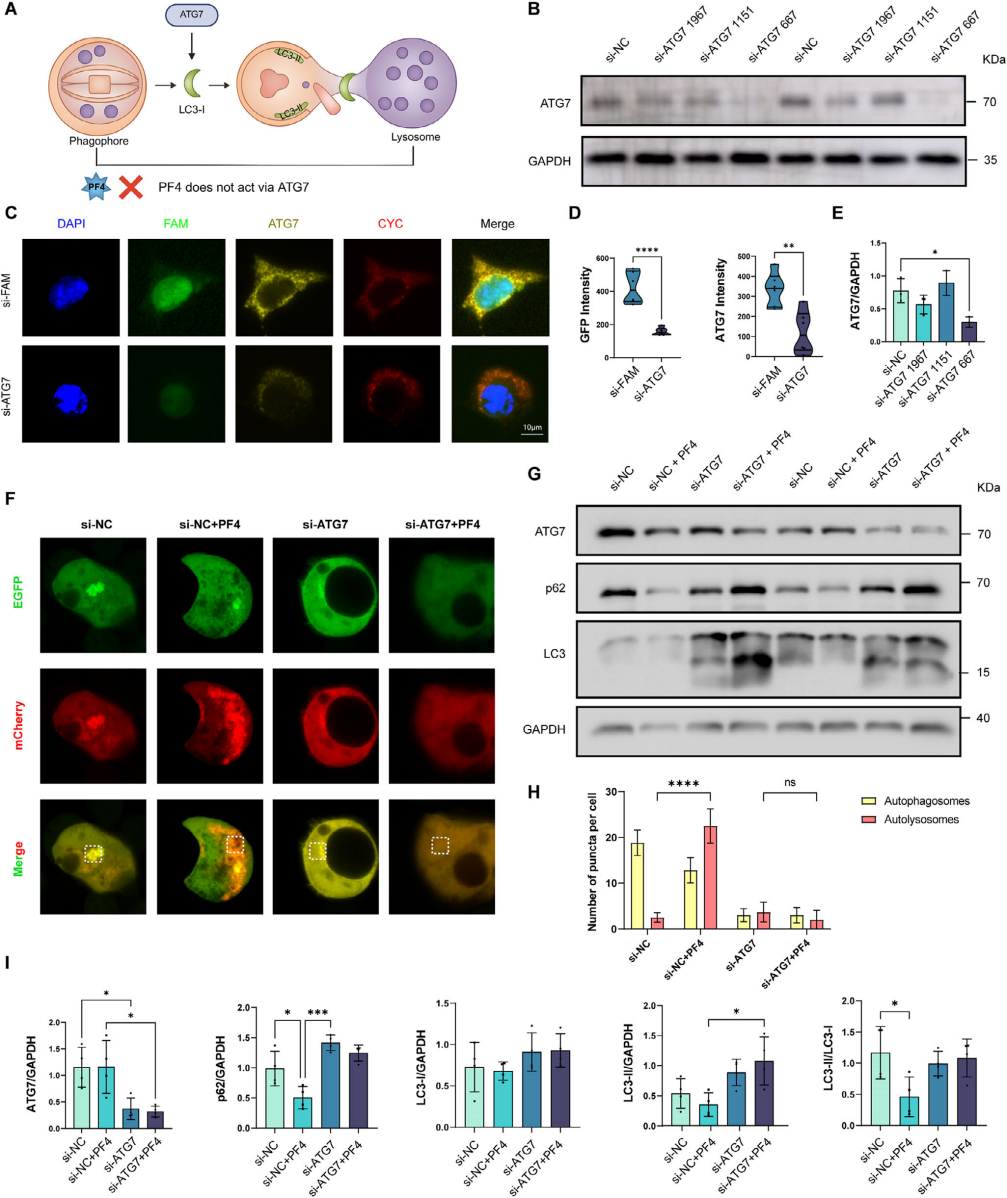
Dependency of PF4‐mediated clearance on the core macroautophagy machinery. (A) Schematic diagram of the autophagy pathway highlighting the crucial role of ATG7 in phagophore elongation and LC3 lipidation. (B,E) Screening of siRNA targeting ATG7. Representative Western blots (B) and the corresponding quantification (E) of ATG7 protein levels to evaluate the knockdown efficiency of three different siRNA sequences. (C,D) Immunofluorescence analysis of knockdown efficiency. Representative confocal images (C) and quantification (D) of ATG7 and GFP fluorescence intensity in si‐FAM (transfection control) and si‐ATG7 transfected cells. (F,H) Direct measurement of autophagic flux dependency using the tandem mCherry‐EGFP‐LC3 reporter. Representative confocal images (F) showing the distribution of EGFP (green) and mCherry (red) signals in cells transfected with si‐NC or si‐ATG7, followed by PF4 treatment. Quantification (H) of the average number of autophagosomes (yellow puncta) and autolysosomes (red‐only puncta) per cell across the indicated conditions. (G,I) Western blot analysis evaluating the functional dependency of PF4 on ATG7. Representative blots (G) of ATG7, p62, and LC3 levels in cells transfected with si‐NC or si‐ATG7 and treated with or without PF4. Densitometric quantification (I) of ATG7, p62, and LC3 turnover normalized to GAPDH. Data are presented as mean ± SEM for bar graphs, and violin plots depict data distribution. Statistical significance was determined using an unpaired two‐tailed Student's t‐test for two‐group comparisons (D), one‐way ANOVA followed by Tukey's post hoc test for multi‐group single‐variable comparisons (E, I), and two‐way ANOVA followed by Sidak's multiple comparisons test for the tandem reporter puncta quantification (H). *p < 0.05, *p* < 0.01, *p < 0.001, ****p < 0.0001; ns, not significant. Scale bars = 10 µm.

To dynamically evaluate this dependency, we utilized the tandem mCherry‐EGFP‐LC3 reporter. While PF4 robustly promoted autolysosome formation in control cells (si‐NC + PF4), ATG7 depletion completely abrogated this effect, resulting in a severe blockade of autophagic flux despite PF4 treatment (Figure 8F,H). These results conclusively indicate that PF4 relies on the canonical core elongation machinery (ATG7), distinguishing it from completely non‐canonical pathways, but utilizes a distinct initiation mechanism.

### 2.9 PF4 Improves Mitochondrial Quality Independently of the PINK1–Parkin Pathway

Prompted by transcriptomic evidence indicating restored mitochondrial function, we assessed mitochondrial health in SOD1 cells. Results showed that SOD1 cells exhibited marked mitochondrial depolarization (reduced TMRE intensity) and an aberrant increase in mitochondria‐lysosome colocalization [30] (Figure 9B–H). Concurrently, the upregulation of protective factors (ATF4, HSP70, COX2) alongside the downregulation of CHOP indicated the activation of an adaptive stress response [31] (Figure 9I–K). This molecular profile suggests that mitochondrial injury in SOD1 cells has not reached an irreversible threshold and remains amenable to therapeutic rescue.

**FIGURE 9:**
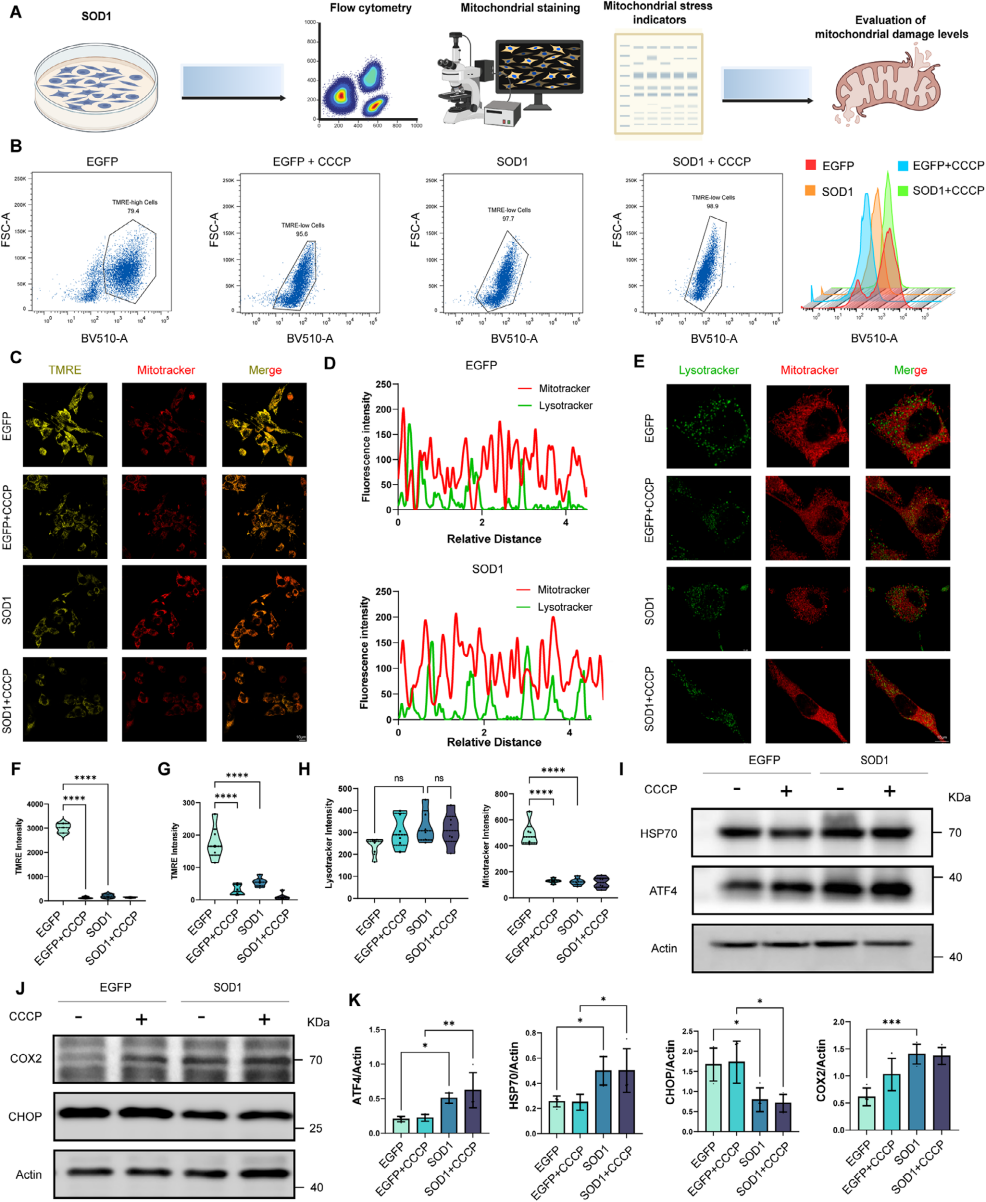
Analysis of mitochondrial dysfunction, mitophagy flux blockade, and stress responses in SOD1 model cells. (A) Schematic workflow of the experimental design evaluating mitochondrial damage, mitophagy, and stress responses. (B, C, F, G) Analysis of mitochondrial membrane potential via TMRE staining. (B) Representative flow cytometry scatter plots and corresponding histogram overlay analyzing TMRE‐positive cell populations in EGFP and SOD1 groups treated with or without CCCP. (C) Representative confocal images showing TMRE (yellow) and MitoTracker (red) distribution. Quantification of mean TMRE fluorescence intensity acquired via flow cytometry (F) and image analysis (G), confirming severe membrane depolarization. (D, E, H) Evaluation of mitophagy flux blockade. (E) Representative confocal images stained with LysoTracker (green) and MitoTracker (red). (D) Corresponding line profile analysis showing the spatial distribution of fluorescence intensities in EGFP (displaying spatial separation of peaks, indicating physiological baseline) and SOD1 (showing abnormal peak entanglement/overlap, reflecting failed clearance) cells. (H) Quantification of LysoTracker and MitoTracker mean fluorescence intensities. (I–K) Western blot analysis of mitochondrial and ER stress markers. Representative immunoblots of (I) HSP70 and ATF4, and (J) COX2 and CHOP in indicated groups. (K) Corresponding densitometric quantification of the stress marker protein levels normalized to Actin. Data are presented as mean ± SEM for bar graphs, and violin plots depict data distribution. Statistical significance was determined using one‐way ANOVA followed by Tukey's post hoc test. *p < 0.05, *p* < 0.01, *p < 0.001, ****p < 0.0001; ns, not significant. Scale bars = 10 µm.

Our findings thus far established that PF4 enhances general autophagic flux and promotes the clearance of SOD1 aggregates. Crucially, our imaging analysis revealed that mutant SOD1 aggregates physically associate with the outer mitochondrial membrane marker TOMM20 (Figure 10A,B). This indicates that PF4‐mediated clearance acts upon a toxic SOD1‐damaged mitochondria macromolecular complex, effectively executing a ‘co‐clearance’ of both pathological protein aggregates and defective organelles. However, the elimination of specific cargos—such as protein aggregates and damaged mitochondria—typically relies on selective autophagy, with mitophagy being a paramount form [32]. We therefore investigated whether PF4 potentiates canonical mitophagy. Unexpectedly, while PF4 upregulated Parkin levels, it downregulated PINK1 expression (Figure 10C–G). The reduction in PINK1 levels is consistent with improved mitochondrial health, as PINK1 is rapidly degraded in healthy mitochondria and only stabilizes upon depolarization. To determine whether the canonical mitophagy pathway mediates the protective effects of PF4, we utilized PINK1 siRNA (Figure 10H–I). Although PINK1 silencing exacerbated mitochondrial damage—confirming the basal protective role of this pathway in SOD1 cells—PF4 retained its ability to mitigate pathology (Figure 10J,K). This confirms that PF4‐mediated enhancement of mitochondrial quality operates independently of the canonical PINK1–Parkin axis.

**FIGURE 10:**
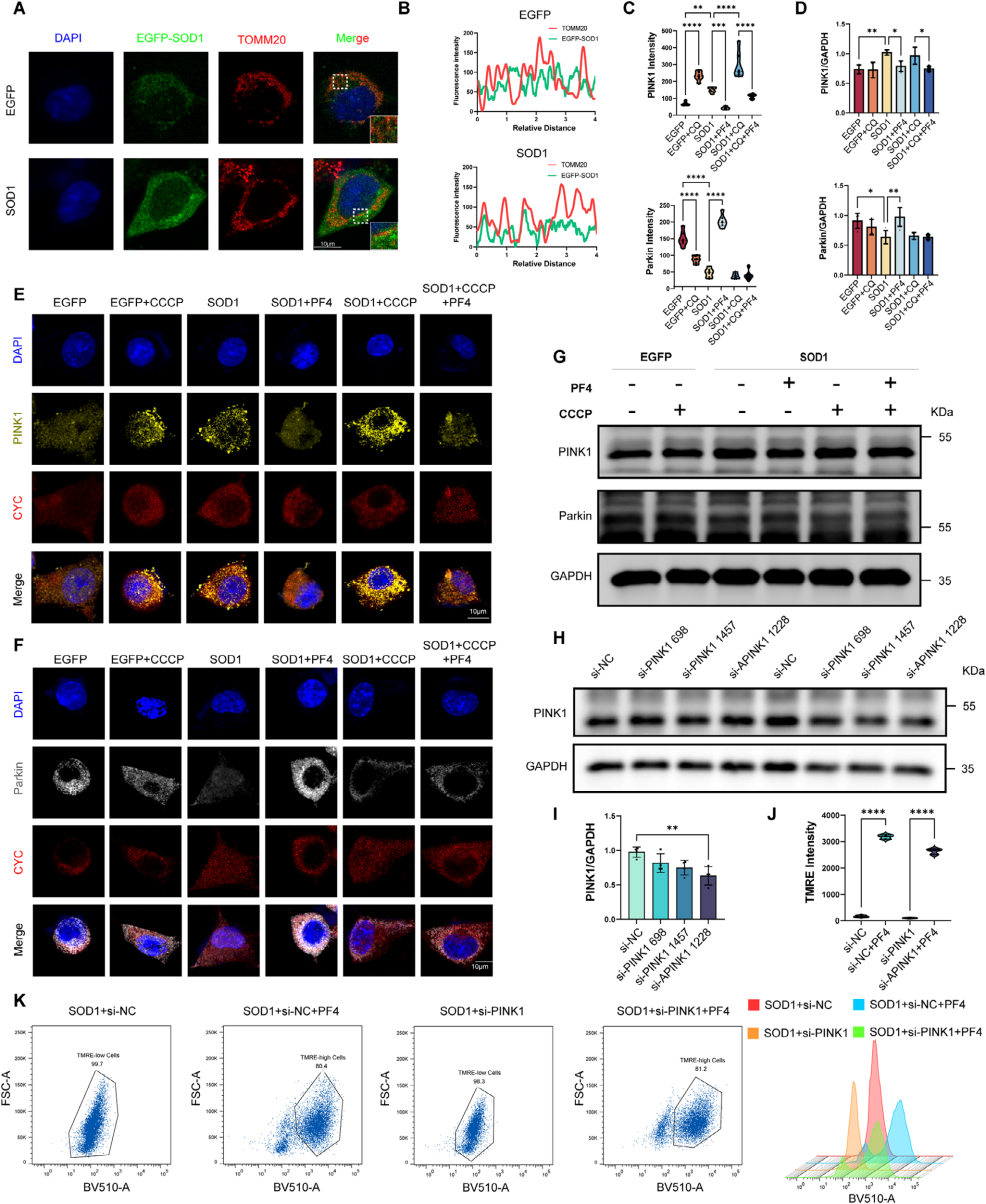
Mutant SOD1 localizes to mitochondria, but PF4‐mediated mitochondrial protection is independent of the canonical PINK1‐Parkin pathway. (A,B) Subcellular localization of mutant SOD1. Representative confocal images (A) showing the spatial relationship between EGFP or EGFP‐SOD1 (green) and the outer mitochondrial membrane marker TOMM20 (red). Nuclei were counterstained with DAPI (blue). (B) Corresponding line profile analysis demonstrating the spatial overlap of EGFP‐SOD1 and TOMM20 fluorescence peaks, indicating pathological recruitment of mutant SOD1 to mitochondria. (C,D) Evaluation of PINK1 and Parkin basal autophagic turnover. Densitometric quantification of PINK1 and Parkin protein levels from Western blots (D) and mean fluorescence intensity from confocal images (C) in cells treated with PF4 and/or the lysosomal inhibitor chloroquine (CQ). (E–G) Assessment of canonical PINK1/Parkin recruitment. Representative confocal images showing the expression and distribution of (E) PINK1 (yellow) and (F) Parkin (white) co‐stained with the mitochondrial marker Cytochrome c (CYC, red) in EGFP and SOD1 cells treated with PF4 or the potent mitochondrial uncoupler CCCP. (G) Representative Western blots of PINK1 and Parkin protein levels across the indicated treatments, comparing PF4‐induced effects against robust CCCP‐induced canonical pathway activation. (H,I) Validation of PINK1 siRNA knockdown. Western blot analysis (H) and corresponding densitometric quantification (I) of PINK1 protein levels to evaluate the knockdown efficiency of three distinct targeted siRNA sequences compared to the negative control (si‐NC). (J,K) Functional dependency assay for PF4‐mediated rescue. Representative flow cytometry scatter plots and histogram overlays (K) evaluating mitochondrial membrane potential via TMRE (BV510‐A) staining in SOD1‐expressing cells co‐transfected with si‐NC or si‐PINK1, and treated with or without PF4. (J) Quantification of mean TMRE fluorescence intensity. The persistent restoration of TMRE signal by PF4 despite PINK1 knockdown establishes a PINK1‐independent rescue mechanism. Data are presented as mean ± SEM for bar graphs, and violin plots depict data distribution. Statistical significance was determined using one‐way ANOVA followed by Tukey's post hoc test. *p < 0.05, *p* < 0.01, *p < 0.001, ****p < 0.0001. Scale bars = 10 µm.

### 2.10 PF4 Enhances Receptor‐Dependent Clearance via OPTN

In selective autophagy, autophagy receptors function as bridges, tethering ubiquitinated cargoes—such as aggregated SOD1 or mitochondria—to LC3‐bound autophagosomes. In the context of neurodegenerative pathology, OPTN and UBQLN2 are two well‐characterized receptors implicated in this process [33, 34].

Given the established independence from the PINK1–Parkin axis, we hypothesized that PF4 potentiates autophagy receptor‐mediated selective clearance (Figure 11A). Protein screening revealed that PF4 induced a specific and significant elevation in the expression of OPTN and UBQLN2, while leaving TAX1BP1 and NDP52 levels unchanged (Figure 11B, C). Immunofluorescence analysis further demonstrated that in SOD1 cells, PF4 treatment significantly enhanced the recruitment of both UBQLN2 and OPTN to damaged mitochondria (marked by TOMM20) (Figure 11D–G).

**FIGURE 11:**
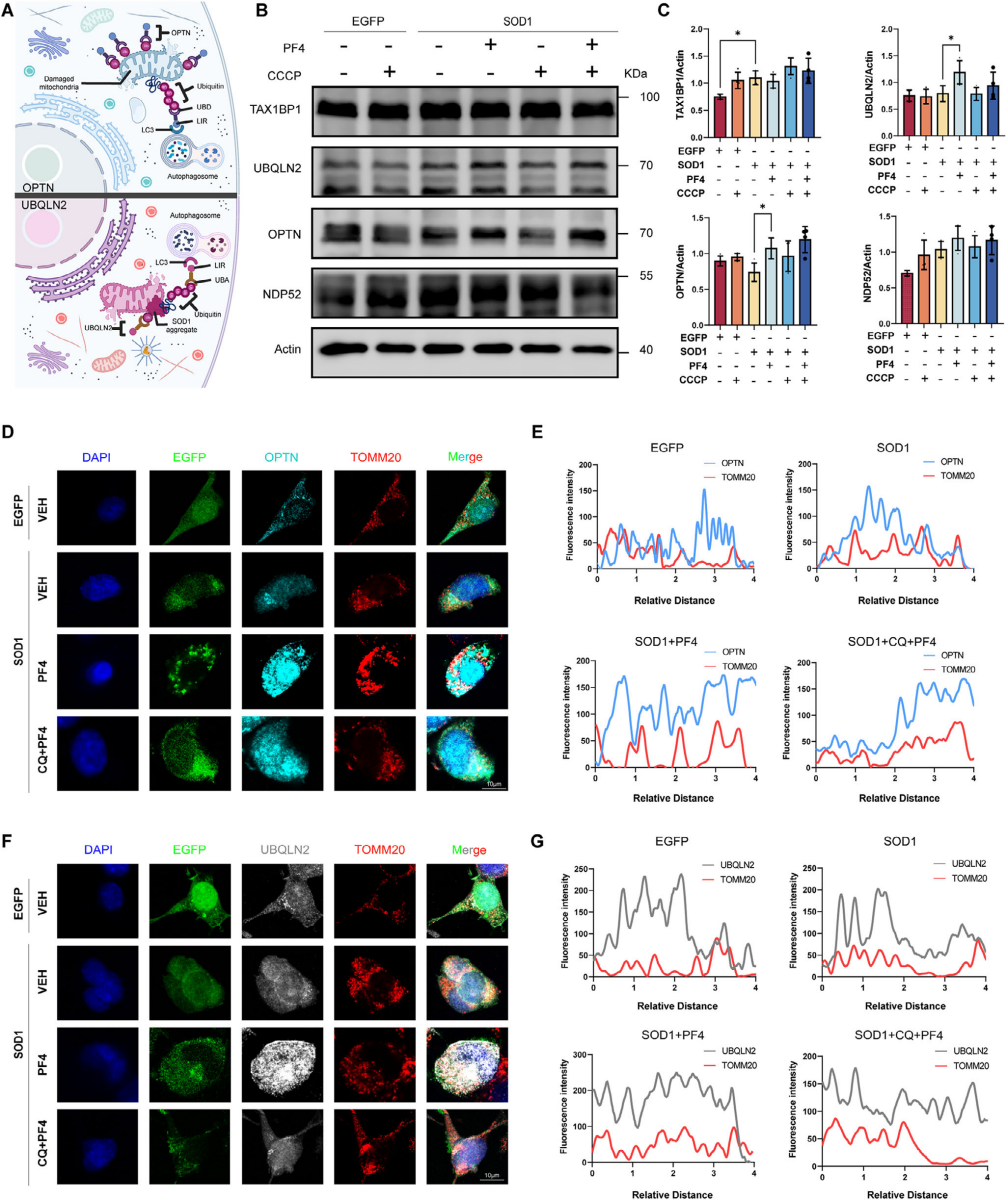
PF4 facilitates the recruitment of autophagy receptors to damaged mitochondria in SOD1‐ALS models. (A) Schematic illustration depicting the proposed mechanism: PF4 restores the recruitment of autophagy receptors (specifically OPTN and UBQLN2) to the mitochondria‐associated degradation machinery, thereby rescuing defective clearance in SOD1‐mutant cells. (B,C) Evaluation of autophagy receptor expression. Representative immunoblots (B) and quantitative analysis (C) showing the expression levels of TAX1BP1, UBQLN2, OPTN, and NDP52 in cells expressing EGFP or SOD1. Cells were treated with PF4 and/or the mitochondrial uncoupler CCCP. Data are normalized to Actin. PF4 treatment significantly rescues the levels of autophagy receptors in SOD1‐expressing cells, comparable to the effect of CCCP. (D,E) Immunofluorescence analysis of OPTN recruitment. Representative confocal images (D) stained for the mitochondrial marker TOMM20 (red), OPTN (cyan), and EGFP/EGFP‐SOD1 (green) across the indicated treatment groups (VEH, PF4, and CQ+PF4). Nuclei were counterstained with DAPI (blue). (E) Corresponding line‐scan fluorescence profiles illustrating the spatial colocalization and overlapping peaks of OPTN and TOMM20 signals. (G,H) Immunofluorescence analysis of UBQLN2 recruitment. Representative confocal images (G) showing the distribution of UBQLN2 (white/grey), TOMM20 (red), and EGFP/EGFP‐SOD1 (green). (H) Corresponding line‐scan profiles demonstrating the spatial overlap of UBQLN2 with mitochondrial markers. Data are presented as mean ± SEM. Statistical significance was determined using one‐way ANOVA followed by Tukey's post hoc test (C). *P* < 0.05. Scale bars = 10 µm.

To pinpoint which receptor is essential for PF4‐mediated protection, we performed functional knockdown experiments. In cells subjected to UBQLN2 knockdown, PF4 fully retained its robust efficacy in clearing SOD1 aggregates (Figure 12F,H,J,K), indicating that UBQLN2 is dispensable for this process. In sharp contrast, OPTN silencing significantly blunted the overall clearance capacity of PF4 compared to the control group (Figure 12G,I). While PF4 still induced a partial reduction of SOD1 by potently upregulating the residual OPTN pool, the marked impairment in its maximal clearance efficiency indicates OPTN as the functional and rate‐limiting mediator (Figure 12C,G,I,K). These data conclusively demonstrate that PF4 promotes the clearance of damaged mitochondria and misfolded SOD1 by selectively potentiating an OPTN‐dependent autophagy pathway.

**FIGURE 12:**
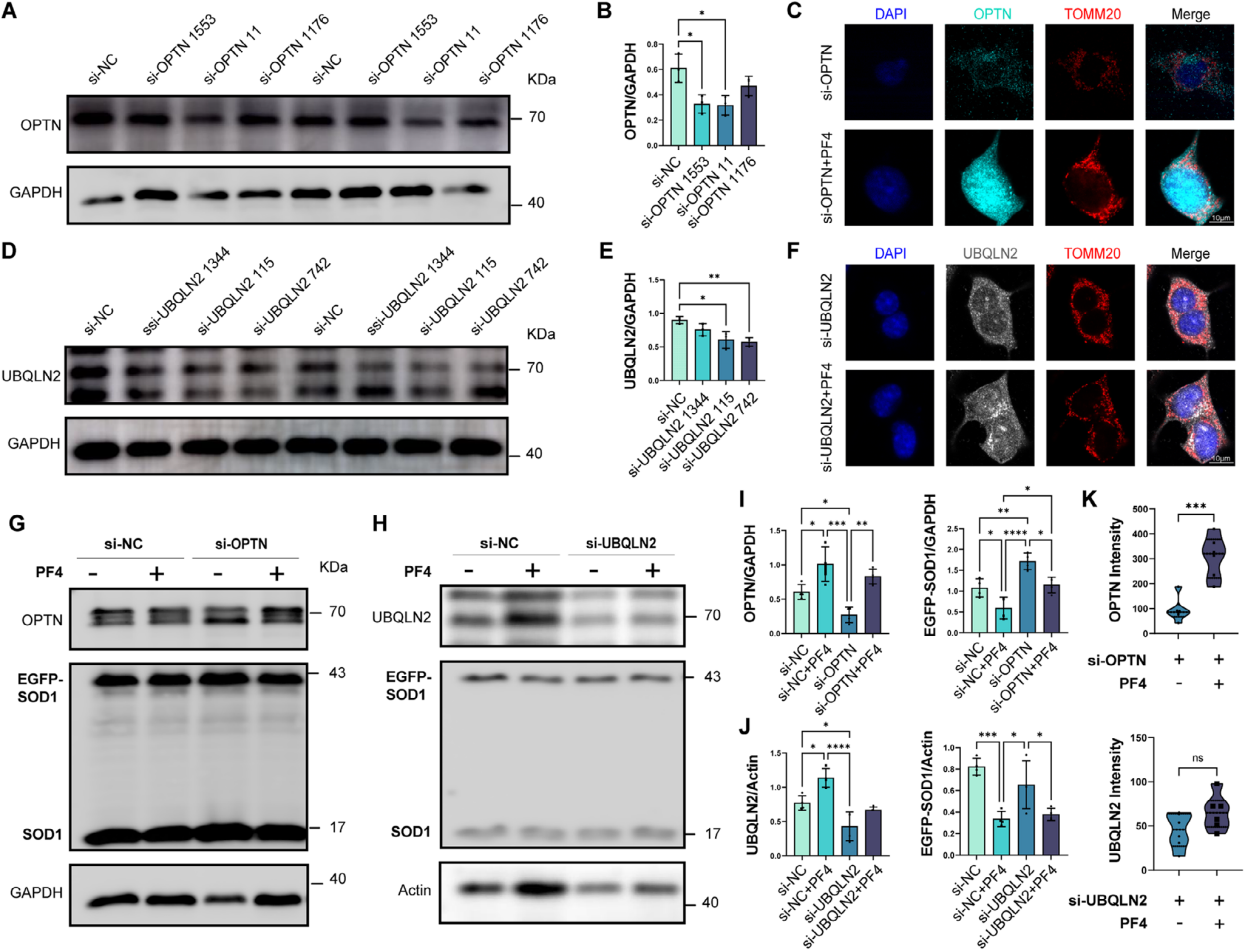
PF4‐mediated clearance of mutant SOD1 depends on OPTN, but not UBQLN2. (A,B) Validation of *Optn* silencing efficiency. Representative immunoblot (A) and quantification (B) of OPTN protein levels following siRNA transfection. (D,E) Validation of *Ubqln2* silencing efficiency. Representative immunoblot (D) and quantification (E) of UBQLN2 protein levels. (C,F,K) Assessment of receptor recruitment. Representative confocal images showing the distribution of OPTN (C, cyan) and UBQLN2 (F, white/grey) co‐stained with TOMM20 (red) in knockdown cells treated with or without PF4. (K) Violin plots quantifying the respective fluorescence intensities. (G,I) Effect of OPTN depletion on SOD1 clearance. Representative immunoblots (G) and corresponding quantification (I) of OPTN and EGFP‐SOD1 protein levels in si‐NC and si‐OPTN cells treated with or without PF4. (H,J) Effect of UBQLN2 depletion on SOD1 clearance. Representative immunoblots (H) and quantification (J) of UBQLN2 and EGFP‐SOD1 protein levels in corresponding knockdown groups. Data are presented as mean ± SEM for bar graphs, and violin plots depict data distribution. Statistical significance was determined using one‐way ANOVA followed by Tukey's post hoc test (B, E, I, J) or unpaired Student's t‐test (K). *p < 0.05, *p* < 0.01, ***p < 0.001, *p* < 0.0001; ns = not significant. Scale bars = 10 µm.

### 2.11 PF4 Signals through the LRP1‐TBK1 Axis to Activate OPTN

Finally, to identify the upstream mechanism by which PF4 initiates this cascade, we first analyzed our transcriptomic dataset to screen established and putative candidate receptors (Figure S8). This unbiased profiling revealed highly significant differential expression in specific targets—notably Lrp1, Gpc1, Sdc1, and Tlr4—providing a data‐driven rationale to focus on LRP1 (Figure 13A and Figure S8). Guided by these screening results, we performed targeted siRNA knockdown (Figure 13B,C). We observed that LRP1 depletion significantly blunted PF4‐induced OPTN phosphorylation (pOPTN) and hindered the subsequent clearance of p62, LC3‐II, and EGFP‐SOD1 (Figure 13D,E). Moreover, evaluation of the downstream signaling interplay revealed that PF4‐induced TBK1 activation (pTBK1) was significantly attenuated in OPTN‐silenced cells, suggesting a mutual functional dependence between TBK1 and OPTN upon PF4 stimulation (Figure 13F,G). Consistent with the biochemical alterations, LRP1 depletion markedly impaired the PF4‐mediated spatial recruitment of OPTN to damaged mitochondria (Figure 13H,I). Collectively, these data complete our mechanistic model, highlighting the LRP1‐TBK1‐OPTN signaling axis as a critical upstream driver for PF4‐mediated selective clearance.

**FIGURE 13:**
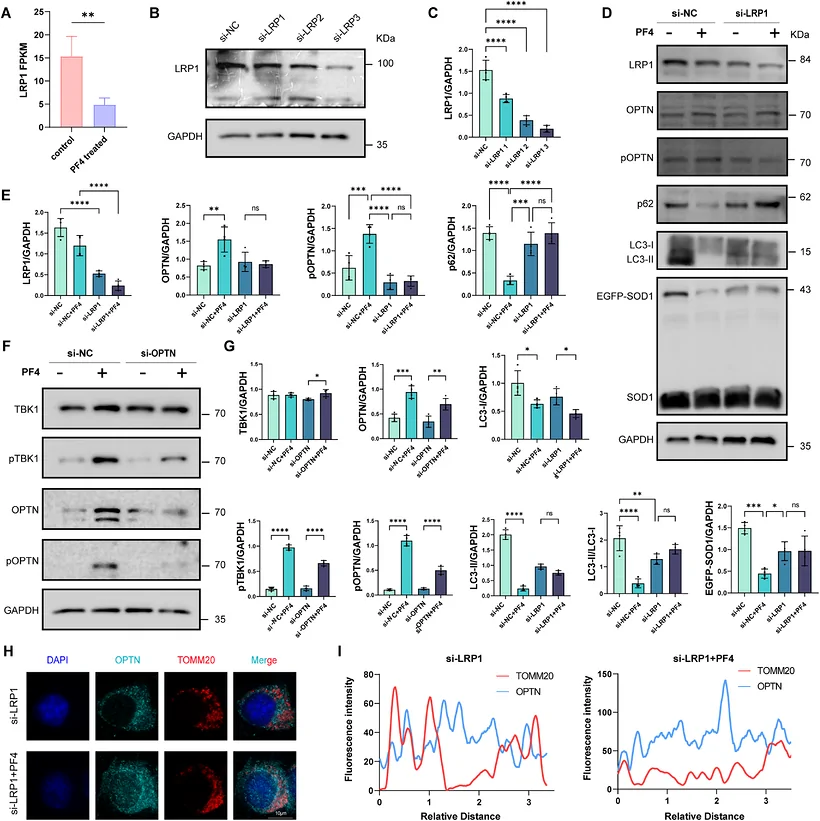
PF4 activates the LRP1‐TBK1‐OPTN signaling axis to promote mitophagy and mutant SOD1 clearance. (A) Quantification of *Lrp1* mRNA expression (FPKM) in control and PF4‐treated cells. (B,C) Validation of *Lrp1* silencing efficiency. Representative immunoblot (B) and quantification (C) identifying the most effective siRNA sequence targeting LRP1. (D,E) LRP1 is essential for PF4‐mediated downstream signaling and SOD1 clearance. Representative immunoblots (D) and corresponding quantification (E) of LRP1, OPTN, phosphorylated OPTN (pOPTN), p62, LC3, and EGFP‐SOD1 protein levels in si‐NC and si‐LRP1 transfected cells treated with or without PF4. (F,G) Interplay between TBK1 activation and OPTN. Representative immunoblots (F) and quantification (G) of TBK1, pTBK1, OPTN, and pOPTN levels in si‐NC and si‐OPTN cells treated with or without PF4. (H,I) LRP1 depletion abolishes PF4‐induced OPTN recruitment. Representative confocal images (H) showing the distribution of OPTN (cyan) and TOMM20 (red) in LRP1‐knockdown cells treated with or without PF4. (I) Corresponding line‐scan profiles confirming the lack of spatial colocalization between OPTN and damaged mitochondria. Data are presented as mean ± SEM. Statistical significance was determined using an unpaired Student's t‐test (A) or one‐way ANOVA followed by Tukey's post hoc test (C, E, G). *p < 0.05, *p* < 0.01, ***p < 0.001, *p* < 0.0001; ns = not significant. Scale bars = 10 µm.

## 3 Disccusion

Here, we identify PF4/CXCL4 as a peripheral neuroprotective modulator that selectively mitigates SOD1‐associated pathology in amyotrophic lateral sclerosis (ALS). Employing an integrative approach that combines population cohort analyses, patient biomarker profiling, and mechanistic dissection in cellular and animal models, we demonstrate that: (1) platelet‐related hematological indices are significantly associated with ALS risk and progression; (2) unbiased multiplex screening reveals that circulating PF4 levels are specifically depleted in patients with ALS, distinguishing it from other neurodegenerative contexts; and (3) PF4 engages the cell surface receptor LRP1 to potentiate lysosome‐dependent autophagic flux, activating a TBK1/OPTN‐mediated and ATG7‐dependent—yet PINK1‐Parkin‐independent—selective autophagy pathway to facilitate the co‐clearance of misfolded SOD1 and damaged mitochondria.

Our findings resonate with a growing body of literature on “peripheral‐central communication,” which posits that blood‐derived systemic factors—including proteins found in young plasma or umbilical cord plasma—can modulate hippocampal plasticity and cognitive function by accessing the brain parenchyma [35, 36, 37]. Contextualizing PF4 as a peripherally derived regulator within this broader physiological framework underscores the biological and translational significance of our findings. In summary, our data unveil a previously unrecognized “platelet‐neuron axis” in ALS pathogenesis and highlight PF4 as a promising therapeutic candidate.

### 3.1 PF4 Bridges Peripheral Hematological Alterations and Central Neurodegeneration

Alterations in systemic immune and hematological states are increasingly recognized as critical modifiers of neurodegenerative diseases, including ALS [38, 39]. Our large‐scale analysis of the UK Biobank cohort unveiled robust associations between platelet indices—specifically platelet count (PLT), plateletcrit (PCT), and platelet distribution width (PDW)—and both ALS incidence and disease progression. This epidemiological evidence suggests that peripheral platelet dynamics may not only reflect but potentially drive central neurodegenerative processes.

Activated platelets are known to release a repertoire of bioactive molecules that orchestrate neuroinflammation, angiogenesis, and neuronal survival [10]. Among these, PF4—an abundant chemokine stored in α‐granules—modulates immune cell activation and tissue repair [17]. Importantly, guided by our unbiased multiplex screening which established PF4 depletion as highly specific to ALS compared to other neurodegenerative conditions such as Alzheimer's and Parkinson's diseases (Figure S2), our comparative analysis revealed that the depletion observed in ALS was not limited to PF4 but also extended to BDNF, whereas VEGF remained stable. This concomitant reduction suggests a potential dysfunction in specific platelet α‐granule cargo release rather than a global secretory failure. However, a key distinction lies in the interaction with aging. While PF4 naturally declines with physiological aging, our age‐adjusted residual analysis demonstrated that the levels in ALS patients consistently deviated from the healthy trajectory, clustering toward the lower limit of the physiological range. This represents a significant negative shift from the expected homeostatic set‐point, implying a disease‐specific critical loss of platelet‐derived trophic support independent of senescence, thereby linking peripheral hematological dysfunction to neuronal vulnerability.

### 3.2 PF4 Selectively Ameliorates SOD1‐Associated Pathology

One of the most intriguing findings of this study is the apparent selective efficacy of PF4 in SOD1‐associated models, in contrast to its lack of observable effect in the context of TDP‐43 or C9orf72 poly(GR30/GA30) pathology under our acute in vitro experimental conditions. This preliminary specificity likely arises from the distinct mechanisms governing proteostatic stress, aggregation, and clearance underlying these ALS subtypes [40, 41, 42, 43]. The misfolding of mutant SOD1 precipitates aberrant protein aggregation and mitochondrial localization leading to injury, processes that are intrinsically linked to autophagic flux [41, 44]. Our data indicate that PF4 specifically restores cellular proteostasis in SOD1 models by enhancing autophagic degradation, rather than by broadly modulating stress responses.

This observation aligns with the findings of Gouel et al., who reported that PF4‐enriched fractions of platelet lysate extended the survival of SOD1 mutant mice [8]. Thus, the in vivo findings by Gouel et al., combined with our explicit cellular selectivity data, collectively suggest that PF4 functions not as a broad‐spectrum anti‐aggregation factor, but as a precision modulator of autophagy‐mediated clearance within specific proteopathic contexts.

### 3.3 Peripheral PF4 Administration Confers Neuroprotection In Vivo

Systemic administration of PF4 significantly improved motor function in hSOD1^G93A^ mice and, although it did not significantly alter disease onset, it markedly extended overall survival (Figure 4). Importantly, our expanded in vivo studies reveal a distinct therapeutic window: while early presymptomatic intervention (P60) achieves optimal mutant SOD1 clearance, a substantial degree of neuroprotective efficacy is still retained even when treatment is delayed until the onset of overt motor symptoms (P90) (Figure S4). This result underscores the functional relevance of circulating PF4 and supports a model wherein peripheral platelets communicate with central neurons via soluble mediators, and highlights its pragmatic translational potential for post‐onset clinical applications.

A critical question concerns the mechanism by which peripheral PF4 accesses the CNS. While ALS pathology is frequently accompanied by blood‐brain barrier (BBB) disruption, which may theoretically provide a conduit for circulating factors [45], our targeted biodistribution assays following extensive transcardial perfusion explicitly confirm that peripherally administered PF4 successfully penetrates and accumulates within the CNS parenchyma (Figure S3). This direct experimental evidence corroborates a recent pivotal study which definitively demonstrated that peripherally administered PF4 is capable of actively crossing the intact BBB to enter the brain parenchyma [46].

### 3.4 PF4 Mediates Neuroprotection via Complementary Anti‐Inflammatory and Synaptogenic Pathways

The neuroprotective efficacy of PF4 appears to be mediated through two complementary pathways, both of which are substantiated by our data in the SOD1 model:

- **Modulation of Neuroinflammation**: As a potent chemokine, a canonical function of PF4 is the remodeling of the immune microenvironment. Consistent with recent reports in aging models [18], PF4 exhibits robust anti‐neuroinflammatory properties. This aligns closely with our observation that PF4 treatment significantly attenuated microgliosis and astrocytosis in the spinal cords of ALS mice (Figure 4N–Q). Crucially, this morphological evidence is further corroborated by our absolute quantitative profiling, which demonstrated that systemic PF4 profoundly blunted the expression cascade of core pro‐inflammatory mediators—including TNF‐α, C1q, CD11b, IL‐1β, and NF‐κB—in the spinal cord microenvironment (Figure S3E).
- **Enhancement of Synaptic Integrity**: Synaptic dysfunction, particularly neuromuscular junction (NMJ) denervation, represents an early pathological hallmark of SOD1‐mutant neurons [47]. PF4 has been demonstrated to directly potentiate synaptic plasticity via NMDAR signaling [46]. This offers a mechanistic rationale for our phenotypic observations: PF4‐mediated enhancement of synaptic plasticity likely underpins the preservation of NMJ integrity and the amelioration of muscle atrophy observed in Figure 5.

Consequently, PF4 does not operate via a solitary mechanism; rather, it mitigates disease progression in hSOD1^G93A^ mice through the dual, synergistic functions of suppressing neuroinflammation and stabilizing synapses

### 3.5 PF4 Reprograms Cellular Homeostasis via Post‐Transcriptional Mechanisms

Our mechanistic dissection of PF4 action reveals an intricate and critical regulatory mode. Transcriptomic analysis indicates that, at baseline, SOD1 model cells exhibit a “hyperactivated” stress state, characterized by the transcriptional upregulation of core autophagy genes (e.g., *ULK1*, *OPTN*, *BNIP3*) (Figure 6E). Strikingly, rather than further amplifying this signal, PF4 treatment induced a concerted transcriptional downregulation of this entire autophagy and mitophagy gene set.

This transcriptional repression (Figure 6E) stands in sharp contrast to our subsequent protein‐level data (Figure 7), which showed that PF4 significantly reduced p62 and LC3‐II levels. Crucially, our dynamic assessment utilizing the tandem mCherry‐EGFP‐LC3 reporter directly visualized the robust maturation of autophagosomes into autolysosomes. This clearance process was entirely abolished by the lysosomal inhibitor chloroquine (CQ), thereby unequivocally confirming the restoration of autophagic flux. Rather than a dichotomy, our data suggests a homeostatic feedback mechanism. In untreated SOD1 cells, the accumulation of aggregates triggers a compensatory upregulation of autophagy transcripts (e.g., OPTN, ULK1) in a futile attempt to clear the burden. PF4 treatment restores the functional autophagic flux at the protein level (indicated by p62 clearance and LC3 turnover). Consequently, the cellular stress signal is dampened, leading to a normalization of autophagy gene transcription. This supports the model that PF4 acts by relieving the blockade in degradation rather than by suppressing transcription directly [48, 49]. This hypothesis is further bolstered by GSEA results revealing a massive, systemic upregulation of the “Ribosome” pathway (Figure 6H), signaling a decisive cellular shift toward translational and post‐transcriptional control.

### 3.6 PF4 Activates an LRP1‐TBK1‐OPTN Selective Autophagy Axis

To delineate the molecular machinery underlying this post‐transcriptional enhancement of autophagic flux, we conducted a systematic pathway dissection. ATG7 serves as a critical upstream node governing autophagy initiation across diverse models [28, 29]. We established that PF4 efficacy relies on the core autophagy machinery, as silencing the pivotal E1‐like enzyme ATG7 completely abrogated PF4‐mediated protection (Figure 8).

A key structural observation in our study is the physical association between mutant SOD1 aggregates and the outer mitochondrial membrane (Figure 10A,B). This spatial linkage suggests the formation of a toxic macromolecular complex, necessitating a ‘co‐clearance’ strategy and prompting our interrogation of canonical mitophagy. However, interrogation of canonical mitophagy revealed a more nuanced landscape. Unexpectedly, PF4 treatment significantly upregulated Parkin levels while concurrently downregulating PINK1 expression (Figure 10). While si‐PINK1 experiments (Figure 10H–K) definitively confirmed that PF4‐mediated neuroprotection is independent of PINK1, the observed upregulation of Parkin may reflect a compensatory response, whereby cells attempt to initiate clearance via Parkin‐dependent, PINK1‐independent mechanisms [50].

This dichotomy—dependence on the core machinery (ATG7) versus independence from the canonical initiation axis (PINK1)—directed our attention toward alternative selective autophagy receptors. OPTN and UBQLN2 are established as critical receptors mediating clearance in neurodegenerative contexts [51, 52]. Indeed, our data confirmed that PF4 significantly enhances the recruitment of both OPTN and UBQLN2 to damaged mitochondria (Figure 11). Crucially, functional knockdown experiments demonstrated that while UBQLN2 is dispensable, OPTN serves as a critical rate‐limiting mediator; OPTN depletion significantly blunted the maximal clearance capacity of PF4, whereas UBQLN2 silencing had no observable effect on SOD1 clearance (Figure 12).

Finally, to complete this mechanistic puzzle, we identified the upstream signaling cascade initiating this receptor activation. Guided by an unbiased transcriptomic screening of potential cell‐surface candidates (Figure S8), we pinpointed LRP1 as the primary transducer. Subsequent targeted interventions revealed that LRP1 depletion, and the resulting disruption of downstream TBK1 activation, markedly impaired PF4‐induced OPTN phosphorylation and its spatial recruitment to mitochondria (Figure 13).

Collectively, these mechanistic data delineate a coherent model: PF4 binds to the cell‐surface receptor LRP1, thereby triggering the TBK1‐pOPTN signaling cascade to potentiate the recognition and co‐clearance of ubiquitinated toxic macromolecular complexes comprising misfolded SOD1 and damaged mitochondria.

### 3.7 Significance and Translational Potential

Our study identifies PF4 as a systemic neuroprotective factor capable of restoring neuronal proteostasis in ALS. By integrating platelet biology with selective autophagy, this work bridges two previously disparate fields: hematological regulation and neurodegeneration. These results open a promising translational avenue: recombinant PF4 or small‐molecule mimetics that potentiate the LRP1‐TBK1‐OPTN signaling cascade may represent innovative therapeutic strategies to facilitate the neuronal clearance of toxic aggregates.

Furthermore, plasma PF4 measurement could serve as a biomarker reflecting the functional status of platelet‐neuron communication, aiding in patient stratification and disease monitoring. Given that OPTN is also implicated in other disorders, such as glaucoma and frontotemporal dementia [53, 54], PF4‐mediated modulation of this pathway may possess broader therapeutic implications transcending ALS.

### 3.8 Limitations and Future Directions

Several limitations warrant consideration. First, although we identified LRP1 as a critical upstream receptor for PF4, the precise structural interaction and co‐receptor dynamics require future investigation. Second, the precise signaling pathways by which PF4 orchestrates the complex dual regulation between transcriptional repression (of autophagy genes) and enhanced protein flux remain to be elucidated. Third, we acknowledge that the observed selectivity of PF4 for SOD1 is currently based on short‐term assays. It remains plausible that chronic PF4 exposure or alternative in vivo dosing regimens could exert subtle neuroprotective effects in TDP‐43 or C9orf72 models, a possibility that warrants comprehensive long‐term evaluation in future studies. Fourth, our biodistribution assays explicitly confirm that peripherally administered PF4 successfully penetrates and accumulates within the CNS (Figure S3), its specific transport rate, CNS half‐life, and precise pharmacokinetics within the pathological context of ALS remain to be fully characterized, necessitating detailed pharmacodynamic studies. Fifth, we acknowledge the substantial translational gap between preclinical efficacy and clinical application, particularly given the profound heterogeneity of sporadic ALS. While systemic administration of recombinant PF4 effectively rescues pathological phenotypes in our animal models, translating these findings into concrete patient benefits will necessitate the development of optimized therapeutic modalities—such as blood‐brain barrier‐penetrant small‐molecule LRP1 mimetics or adeno‐associated virus (AAV)‐mediated gene therapy. Future rigorous clinical trials will be indispensable for stratifying specific patient subgroups (e.g., those with profound PF4 deficiency) most likely to respond to this targeted intervention. Future work employing PF4 receptor knockouts and single‐cell transcriptomics will be instrumental in clarifying how PF4 remodels cellular networks within the ALS microenvironment.

## 4 Materials and Methods

### 4.1 Human Studies

#### 4.1.1 UK Biobank Cohort

This study utilized data from the UK Biobank (UKB) cohort, which enrolled 502 357 participants aged 40–69 years at baseline. ALS diagnoses were identified via ICD‐10 code G12.2. Participants with a baseline diagnosis of ALS, comorbid autoimmune disorders, missing hematological data, or incomplete follow‐up records were excluded.

#### 4.1.2 Patient Recruitment and Ethics

This study was approved by the Ethics Committee of Wenzhou Medical University, and informed consent was obtained from all participants. We recruited 40 patients with sporadic ALS and 40 age‐ and sex‐matched healthy controls from The First Affiliated Hospital of Wenzhou Medical University. ALS diagnosis was established according to the revised El Escorial criteria.

To ensure the accuracy of platelet‐derived factor quantification, stringent exclusion criteria were applied: (1) presence of other neurological or severe systemic diseases; (2) active infection or autoimmune disorders; and (3) use of anti‐platelet (e.g., aspirin, clopidogrel) or anti‐coagulant medications within two weeks prior to sampling, as well as any history of hematological disorders.

### 4.2 Animal Models

#### 4.2.1 Transgenic Mice

Transgenic hSOD1^G93A^ mice [B6SJL‐Tg(SOD1^G93A^)1Gur/J] and their wild‐type (WT) littermates were purchased from The Jackson Laboratory [55, 56]. All animal experimental procedures complied with the Regulation on the Administration of Laboratory Animals (Ministry of Health, People's Republic of China) and were approved by the Animal Ethics Committee of Wenzhou Medical University.

#### 4.2.2 Housing and Husbandry

Mice were housed in a specific pathogen‐free (SPF) facility under controlled temperature (22–24°C) and humidity (40–60%) conditions, with a 12‐h light/12‐h dark cycle and ad libitum access to sterile water and standard pellet chow. Both male and female mice were utilized, with sex balanced across analyses. Genotyping was performed via PCR analysis of tail DNA using specific primers targeting the human *SOD1* gene (sequences are listed in the Key Resources Table).

### 4.3 Cell Lines

#### 4.3.1 NSC‐34 and HEK293 Cells

Mouse neuroblastoma‐spinal cord hybrid NSC‐34 cells [57] and human embryonic kidney (HEK293) cells [58] were maintained in high‐glucose DMEM supplemented with 10% fetal bovine serum (FBS) and 1% penicillin‐streptomycin (P/S). Cells were cultured in a humidified incubator at 37°C with 5% CO_2_. All cell lines were routinely tested for mycoplasma contamination using the MycoProbe kit and confirmed to be negative.

### 4.4 Analysis of UK Biobank Data

Platelet‐related indices—including platelet count (PLT), plateletcrit (PCT), platelet distribution width (PDW), and mean platelet volume (MPV)—were extracted and analyzed. Among the initial 502 357 UK Biobank participants, we first excluded 78 individuals with prevalent ALS at baseline. Subsequently, participants with missing follow‐up data (N = 1297) or missing baseline hematological indices (N = 32,545) were excluded. Importantly, to prevent confounding bias, we further excluded 20,746 individuals with comorbid autoimmune disorders (representing ∼4.1% of the eligible cohort), as systemic autoimmunity independently alters baseline platelet indices. Following these rigorous exclusions, the final cohort included in the main analysis comprised 447 691 participants. We utilized Cox proportional hazards models to evaluate the associations of these parameters with ALS incidence and progression, performing both overall and stratified analyses. Models were adjusted for potential confounders, including age, sex, body mass index (BMI), smoking status, alcohol consumption, and the Townsend deprivation index.

### 4.5 Human Plasma Collection and Cytokine Quantification

The collection and use of human clinical samples in this study were approved by the Ethics Committee of the First Affiliated Hospital of Wenzhou Medical University (Approval No. KY2024‐R267). Written informed consent was obtained from all participants prior to their enrollment and sample collection. Peripheral venous blood was collected into tubes containing EDTA (or sodium citrate) as an anticoagulant to prevent ex vivo platelet activation. Plasma was isolated via centrifugation at 2 500 × g for 20 min at 4°C. Plasma aliquots were immediately snap‐frozen and stored at ‐80°C until analysis. The circulating concentrations of platelet‐derived cytokines—specifically PF4/CXCL4, BDNF, and VEGF—were quantified using multiplex ELISA kits strictly in accordance with the manufacturers' instructions (see Key Resources Table).

Normality was assessed via Shapiro‐Wilk tests. Data are presented as mean ± SEM for normally distributed variables or median with interquartile range (IQR) for non‐parametric data. Group comparisons were conducted using unpaired two‐tailed t‐tests or Mann‐Whitney U tests, and distributions were visualized using Raincloud plots. Correlations were assessed using Pearson coefficients. To isolate disease‐specific effects from physiological aging, we calculated age‐adjusted residuals based on a linear regression model derived from healthy controls. Diagnostic performance was quantified by ROC curve analysis, with AUC comparisons performed using the DeLong test. Statistical significance was defined as p < 0.05.

### 4.6 Plasmid Construction

The pTRE3G‐mCherry‐BI‐EGFP series plasmids (wild‐type, SOD1^G93A^, and TDP‐25‐PrLD variants) were kindly provided by Hebei Medical University [59, 60]. To construct dipeptide repeat (DPR) expression plasmids, synthetic DNA fragments encoding (GA)_30_ or (GR)_30_ were subcloned into the GV144 vector (GeneChem) to generate pCMV‐EGFP‐(GA)_30_ and pCMV‐EGFP‐(GR)_30_ [61, 62]. Additionally, the tandem fluorescent reporter plasmid for monitoring autophagic flux, pEX‐3‐mCherry‐EGFP‐LC3, was purchased from GenePharma (Shanghai, China). All constructs were verified via Sanger sequencing.

To preclude spectral interference and fluorescence crosstalk when simultaneously assessing autophagic flux with the tandem mCherry‐EGFP‐LC3 reporter, we employed a non‐fluorescent (unlabeled) SOD1^G93A^ expression plasmid for these specific co‐transfection experiments, effectively eliminating any potential signal overlap between the SOD1 cargo and the LC3‐based flux probe.

### 4.7 Cell Transfection and In Vitro Models

#### 4.7.1 Transfection

Plasmids were transfected into cells at 70%–90% confluence using Lipofectamine 2000 in Opti‐MEM reduced‐serum medium. For siRNA‐mediated gene knockdown (targeting *ATG7*, *OPTN*, *UBQLN2*, *PINK1*), cells were transfected with 20 µM siRNA or control siRNA using GA‐RNA Transfection Reagent and harvested 48–96 h post‐transfection.

#### 4.7.2 Stable Cell Lines

NSC‐34 cells transfected with the pTRE3G series plasmids were selected with G418 (1000 µg/mL) for 2 weeks and subsequently maintained at 200 µg/mL [63].

#### 4.7.3 Acute Injury Models

Autophagy impairment models were established by treating cells with 100 µM chloroquine (CQ) for 2 h [64]. Mitochondrial damage models were induced by treatment with 10 µM CCCP for 6 h [65].

### 4.8 Cell Viability Assay

Cell viability was assessed using the CCK‐8 kit. Cells were seeded in 96‐well plates (3000 cells/well) and, following 24 h of serum‐free starvation, were treated with varying concentrations of PF4 (0–500 ng/mL) for 24 h. CCK‐8 solution (10 µL/well) was added, and cells were incubated at 37°C for 2–4 h. Absorbance was measured at 450 nm using a Varioskan Flash microplate reader.

### 4.9 Flow Cytometry for Mitochondrial Membrane Potential

Cells were harvested via trypsinization, resuspended in TMRE working solution (0.5–1 × 10^6^ cells/mL), and incubated at 37°C for 30 min in the dark. CCCP‐treated cells served as positive controls. Samples were filtered through a 40‐µm strainer and immediately analyzed for changes in mitochondrial membrane potential (MMP) using a flow cytometer.

### 4.10 Animal Treatment and Grouping

#### 4.10.1 Early Intervention Cohort (P60)

Twenty hSOD1^G93A^ mice were randomly assigned to PF4 treatment or vehicle control groups (n = 10 per group), with 10 wild‐type mice serving as negative controls. Randomization was performed using a computer‐generated random number sequence to allocate animals to each group, and investigators conducting behavioral and histological assessments were blinded to group identity. Starting at postnatal day 60 (P60), the treatment group received recombinant mPF4 (5ug/ml, 100 µL/dose (∼25–33 µg/kg), PROSPEC, CHM‐245) via tail vein injection every 72 h for a total of 8 injections (over 24 days) [66]. The sample size was determined based on prior studies using the hSOD1^G93A^ model that demonstrated robust and reproducible behavioral and pathological differences with group sizes of 8–12 animals. Thus, a group size of 10 was selected a priori to provide adequate statistical power while minimizing animal use, consistent with ARRIVE guidelines. Individuals with extremely poor health status were excluded from the experiment.

#### 4.10.2 Post‐Onset Intervention Cohort (P90)

To specifically evaluate the therapeutic window of PF4, an independent supplementary cohort was established. Eighteen mice were randomly allocated into three groups (*n* = 6 per group: WT, hSOD1^G93A^ + Vehicle, and hSOD1^G93A^ + PF4). Starting at postnatal day 90 (P90)—a defined timepoint corresponding to the onset of overt motor symptoms—the mice received PF4 or vehicle treatments following the identical dosage and administration route described above. While this supplementary cohort utilized a modest sample size due to strict transgenic breeding time constraints, it was sufficient to provide robust statistical power for validating post‐onset phenotypic and molecular alterations.

### 4.11 Blood Collection and Plasma Preparation

To monitor longitudinal peripheral PF4 dynamics, blood samples were harvested from mice at designated time points (postnatal days 70, 90, and 110). Mice were deeply anesthetized with isoflurane, and whole blood was collected from the retro‐orbital sinus using capillary tubes pre‐coated with ethylenediaminetetraacetic acid (EDTA). To completely prevent mechanical stress‐induced platelet activation and subsequent artificial release of intracellular PF4 granules, blood was immediately drained into tubes containing a 10% volume of EDTA anticoagulant and mixed by gentle inversion.

Platelet‐poor plasma (PPP) was isolated using a rigorous sequential double‐centrifugation protocol. Briefly, whole blood was initially centrifuged at 2000 × g for 15 min at 4°C to separate cellular components. The upper plasma layer was carefully transferred to a clean microcentrifuge tube, avoiding the buffy coat interface, and subjected to a second high‐speed centrifugation at 10 000 × g for 10 min at 4°C to fully precipitate residual platelets and microparticles. The purified supernatant plasma was immediately aliquoted, snap‐frozen in liquid nitrogen, and stored at −80°C until further analysis.

### 4.12 In Vivo Biodistribution and Tissue Collection

To evaluate the capability of peripherally administered PF4 to cross the blood‐brain barrier (BBB) and reach the central nervous system (CNS) parenchyma, an in vivo biodistribution assay was performed. Age‐ and sex‐matched hSOD1^G93A^ mice received a single intravenous (i.v.) or intraperitoneal (i.p.) injection of recombinant mouse PF4 (500 µg/kg) or vehicle. At exactly 10 min post‐injection, the mice were deeply anesthetized. To assess systemic drug levels, blood was first drawn carefully from the right ventricle using an EDTA‐coated syringe and immediately centrifuged at 2000 × g for 15 min at 4°C to isolate the plasma supernatant. Crucially, immediately following blood collection, the mice were subjected to extensive transcardial perfusion with ice‐cold PBS (without fixative) for at least 5 min to eliminate any contaminating circulating PF4 from the tissue microvasculature. Perfusion was deemed complete when the liver blanched and the efferent fluid from the right atrium ran clear. Subsequently, the brain parenchyma, spinal cord, and liver were rapidly dissected, rinsed in cold PBS, flash‐frozen in liquid nitrogen, and stored at −80°C to preserve native protein epitopes for downstream quantitative assays.

### 4.13 Protein Extraction and Quantitative ELISA

For tissue analyses, frozen spinal cords and brain parenchyma were mechanically homogenized in RIPA Lysis Buffer (Beyotime, P0013B) supplemented with a protease and phosphatase inhibitor cocktail (GLPBIO). The homogenates were centrifuged at 14,000 × g for 15 min at 4°C, and the total protein concentrations of the clear supernatants were strictly determined using a BCA Protein Assay Kit (GLPBIO, GK10009) to ensure accurate downstream normalization. Concurrently, the previously isolated plasma samples were thawed on ice and appropriately diluted.

Absolute protein levels were quantified using high‐sensitivity enzyme‐linked immunosorbent assay (ELISA) kits strictly according to the manufacturers' instructions. For the human clinical cohort, absolute biomarker concentrations in serum/plasma were measured using specific kits for human PF4/CXCL4 (R&D Systems, DPF40), human BDNF (Abcam, ab212166), and human VEGF‐A (Abcam, ab222510). For the in vivo mouse models, PF4 concentrations in systemic plasma were measured using the Mouse CXCL4/PF4 ELISA Kit (Abcam, ab100735). For the neuroinflammatory profiling of the spinal cord, specific kits were utilized to quantify TNF‐α (Abcam, ab208348), C1q (Abcam, ab270884), CD11b (Cusabio, CSB‐E17417m), IL‐1β (Abcam, ab197742), and total NF‐κB p65 (Abcam, ab176647). To account for variations in tissue mass and extraction efficiency, the absolute target concentrations derived from the standard curves were normalized to the total protein content of each respective sample. Final tissue concentrations were expressed as picograms of target protein per milligram of total protein (pg/mg), whereas systemic plasma concentrations were expressed as nanograms per milliliter (ng/mL).

### 4.14 Behavioral Assessments

Behavioral testing commenced at postnatal day 90 (P90) and was conducted weekly in a low‐noise environment.

#### 4.14.1 Rotarod Test

Mice were placed on an accelerating rotarod (5–40 rpm over 300 s). The latency to fall was recorded. Three trials were performed per session, and the maximum value was retained.

#### 4.14.2 Neurological Score

A standardized 5‐point scale was employed (5 = normal; 1 = complete paralysis).

#### 4.14.3 Grip Strength and Hanging Test

Limb muscle strength was measured using a grip strength meter; grip endurance was assessed via the inverted wire hanging test (cutoff: 90 s).

#### 4.14.4 Gait Analysis

Stride length and the trajectory of hindlimb footprints were measured.

### 4.15 Disease Onset and Progression

Disease onset was defined retrospectively as the day of peak body weight, typically coinciding with a decline in the neurological score to 4 (indicating mild tremors or reduced hindlimb splay reflex). The primary endpoint for survival analysis was defined as either death or the humane endpoint of complete hindlimb paralysis, at which point animals were euthanized according to institutional guidelines.

### 4.16 Tissue Collection and Preparation

Ten days post‐disease onset, four mice per group were randomly selected for tissue harvesting.

#### 4.16.1 For Biochemistry

Following anesthesia, spinal cords (cervical, thoracic, and lumbar segments) were rapidly dissected, rinsed with saline, and flash‐frozen in liquid nitrogen.

#### 4.16.2 For Histology

Mice were transcardially perfused with PBS followed by 4% PFA. Spinal cords were post‐fixed overnight, cryoprotected in 30% sucrose, and embedded in O.C.T. compound. Gastrocnemius and tibialis anterior muscles were embedded directly and flash‐frozen in isopentane pre‐cooled with liquid nitrogen.

### 4.17 Immunofluorescence and Live Cell Imaging

#### 4.17.1 Cells

Cells were fixed with 4% PFA, permeabilized with Triton X‐100, blocked, and incubated with primary antibodies (see Key Resources Table) overnight at 4°C. Secondary antibodies were applied the following day, and nuclei were counterstained with DAPI. For live‐cell imaging, organelles were labeled with Mito‐Tracker Deep Red FM and Lyso‐Tracker Red, utilizing Hoechst 33342 for nuclear staining.

#### 4.17.2 Spinal Cord

Twenty‐micron (20 µm) floating sections were blocked and incubated with primary antibodies (e.g., anti‐CTSD, anti‐NeuN) followed by corresponding fluorescent secondary antibodies. Z‐stack images were acquired using a Leica confocal microscope, and maximal intensity projections were generated.

### 4.18 Western Blotting

Protein concentrations of tissue or cell lysates were determined via the BCA assay. Samples underwent SDS‐PAGE and were transferred onto PVDF membranes. Membranes were blocked and incubated overnight with specific primary antibodies (SOD1, TDP‐43, OPTN, LC3B, p62, UBQLN2, GFAP, Iba‐1, etc.; details listed in Key Resources Table). Blots were visualized using HRP‐conjugated secondary antibodies and ECL chemiluminescence reagents. Band intensity was analyzed using Image Lab software and normalized to GAPDH or β‐Actin.

### 4.19 Muscle Histology and NMJ Analysis

#### 4.19.1 H&E and NADH‐TR

Muscle cryosections (14 µm) were subjected to H&E staining (to assess myofiber atrophy) and NADH‐TR enzyme histochemistry (to evaluate mitochondrial functional distribution). Myofiber cross sectional area (CSA) was quantified using Fiji software.

#### 4.19.2 NMJ Staining

Longitudinal muscle sections (30 µm) were fixed in methanol. Nerve terminals were labeled with anti‐SV2 and anti‐SMI‐32, while post‐synaptic receptors were visualized using Alexa Fluor 647‐α‐Bungarotoxin. Colocalization at the neuromuscular junction was analyzed via confocal imaging.

### 4.20 Motor Neuron Counting (Nissl Staining)

Spinal cord cryosections were stained with cresyl violet (Nissl). Motor neurons in the ventral horn with a diameter >8 µm and a distinct nucleolus were counted by an independent investigator blinded to the experimental groups.

### 4.21 RNA‐Seq and Bioinformatics

Total RNA was extracted using TRIzol reagent, and library construction and sequencing were performed by Majorbio (Shanghai, China). A subset of data was obtained from the GEO database (GSE158264). Differential expression analysis was performed using the “limma” package in R [67]. Functional enrichment analyses (GO, KEGG, GSEA) were conducted using the “clusterProfiler” package [68, 69].

### 4.22 Transmission Electron Microscopy (TEM)

Mice were perfused with fixative (2% PFA + 2.5% glutaraldehyde). Sciatic nerves were harvested, post‐fixed, stained with osmium tetroxide, dehydrated, and resin‐embedded. Ultrathin sections (60–90 nm) were prepared and double‐stained with uranyl acetate and lead citrate [70]. Myelin and axonal ultrastructure were examined using a JEM‐F200 TEM. Damaged mitochondria were defined by swelling, cristae fragmentation, or membrane disruption [71].

### 4.23 Quantification and Statistical Analysis

Statistical analyses were performed using GraphPad Prism 9.5.0 and R (v4.4.1). Normally distributed data are presented as mean ± SD. Comparisons between two groups were analyzed using Student's t‐test, while multi‐group comparisons utilized ANOVA followed by Šidák or Tukey post hoc tests. Non‐normal data were analyzed using Mann‐Whitney U or Kruskal‐Wallis tests. Pearson's coefficient was used for correlation analysis. ROC curve analysis was employed to assess biomarker performance. P < 0.05 was considered statistically significant. All hSOD1^G93A^ mouse experiments and quantitative analyses were conducted under double‐blind conditions. Specific sample sizes (n) and statistical methods are detailed in the respective figure legends. All schematic diagrams were created using BioRender. Statistical plots were generated using Chiplot, and final Figure were assembled in Adobe Illustrator.

## Author Contributions

Q.X., D.F., and B.D.: conceptualization and Writing – review & editing. Q.X., Y.L., H.X., and W.J.: methodology. Q.X., Y.L., H.X., W.J., Y.Z., M.H., Y.P., and C.J.: investigation. Y.Z., H.Y., and K.L.: data curation. H.Y. and K.L.: Formal analysis. Q.X. and H.X.: visualization. D.F., B.D., and X.S.: supervision. Q.X.: Writing – original draft.

## Funding

This work was supported by the Natural Science Foundation of Zhejiang Province (grants ZCLY24H0903 to B.D.) and Hebei Key Laboratory of Neurology (No. 2025L‐C7 to B.D.).

## Conflicts of Interest

The authors declare no conflict of interest.

## Supporting information




**Supporting File**: advs76778‐sup‐0001‐SuppMat.docx.

## Data Availability

The processed RNA‐seq data (including quantification matrices) supporting the findings of this study have been deposited in Mendeley Data for peer review purposes (https://data.mendeley.com/preview/xx2jzy64hp?a=64e82365‐fcc7‐45aa‐a025‐738a3e7b4068). The raw sequencing data have been deposited in the NCBI Gene Expression Omnibus (GEO) under the accession number GSE338930. UK Biobank data: This research has been conducted using the UK Biobank Resource under Application Number [108832]. The data are available from the UK Biobank (http://www.ukbiobank.ac.uk/) upon successful registration and application. Microscopy and Western blot data: All original western blot images, microscopy data, and other supporting data reported in this paper will be shared by the lead contact upon request.
