# Supplementary material for: A Blood‐Derived Factor Rescues ALS: Platelet Factor 4 Activates OPTN‐Dependent Autophagy to Clear SOD1 Aggregates Independently of PINK1
Source: Adv Sci (Weinh). 2026 Jul 23;13(58):e76778. doi: 10.1002/advs.76778 (PMC13393263; doi:10.1002/advs.76778)
Supplement: Supplementary file 1 [file ADVS-13-e76778-s001.docx]

Supplementary Materials for

**A Blood-Derived Factor Rescues ALS: Platelet Factor 4 Activates OPTN-Dependent Autophagy to Clear SOD1 Aggregates Independently of PINK1**

Qingjian Xie *et al.*

*Corresponding author. Email: dbb2013@163.com

**This PDF file includes:**

Figs. S1 to S12

Tables S1 to S4

**Supplemental Figures**

**Graphical abstract**

**Figure S1.** Association analysis of hematological parameters with ALS risk.

**Figure S2.** Multiplex profiling of peripheral blood biomarkers demonstrates the disease-specific reduction of PF4 in ALS.

**Figure S3.** Plasma PF4 dynamics, BBB penetrance of systemic PF4, and its robust anti-inflammatory efficacy in the central nervous system.

**Figure S4.** PF4 administration facilitates hSOD1 clearance and ameliorates disease progression in hSOD1^G93A^ mice.

**Figure S5.** Transcriptional analysis and pathway enrichment in SOD1-mutated iPSC-derived motor neurons.

**Figure S6.** Advanced functional enrichment visualization of DEGs in SOD1-mutated iPSC-motor neurons.

**Figure S7.** Assessment of basal autophagic flux in SOD1-expressing cells.

**Figure S8.** Transcriptomic screening of potential upstream receptors for PF4.

**Figure S9.** Original full-length Western blot images supporting the main text figures.

**Figure S10.** Original full-length Western blot images supporting the main text figures.

**Figure S11.** Original full-length Western blot images supporting the main text figures.

**Figure S12.** Single-color imaging controls confirm the absence of fluorescence bleed-through in confocal microscopy.

**Supplemental Tables**

**Table S1.** Key resources table

**Table S2.** Demographic characteristics and biomarker levels stratified by age

**Table S3.** Demographic characteristics and multiplex biomarker profiles of the expanded cross-sectional cohort

**Table S4.** ROC analysis results of Platelet-related factors for ALS vs HC

**Graphical abstract**

**Figure S1. Association analysis between hematological parameters and ALS risk.** **(D)** Schematic workflow of the epidemiological and statistical analysis pipeline. **(A)** Circular heatmap illustrating the correlation between baseline blood cell indices and selected covariates in the study cohort (n = 447,691). The color scale represents the correlation coefficient, with blue indicating a negative correlation and red indicating a positive correlation. **(B)** Correlation network plot evaluating the association between incident ALS diagnosis and individual hematological parameters. Node size represents the -log10(P-value), while red and blue edges denote positive and negative correlations, respectively. Line thickness indicates correlation strength (|R|). **(C)** Heatmap of Hazard Ratios (HR) derived from Cox proportional hazards regression models evaluating the risk of ALS. Model 1 is adjusted for basic demographic factors, and Model 2 is further fully adjusted for body mass index (BMI), smoking status, alcohol consumption, and the Townsend deprivation index. The color gradient reflects the HR (red, HR > 1.0; blue, HR < 1.0). Statistical significance is indicated by *P < 0.05, P < 0.01.

**Figure S2. Multiplex profiling of peripheral blood biomarkers demonstrates the disease-specific reduction of PF4 in ALS.** **(A)** Schematic overview of the expanded cross-sectional cohort study. Plasma samples were collected from rigorously matched cohorts of healthy controls (HC), amyotrophic lateral sclerosis (ALS), Alzheimer's disease (AD), and Parkinson's disease (PD) patients (n = 60 per group, strictly adjusted for age, sex, and other demographic factors) for multiplex ELISA analysis of 10 platelet-associated and neurotrophic factors. **(B)** Volcano plot illustrating the differentially expressed circulating factors specifically between the ALS and HC cohorts. Red and blue dots represent significantly up-regulated and down-regulated biomarkers, respectively (thresholds: P < 0.05). **(C)** Violin plots with integrated box plots displaying the absolute serum concentrations of the 10 target biomarkers across the four cohorts. Notably, the significant depletion of PF4 is highly specific to the ALS group, whereas its levels remain relatively stable in both the AD and PD cohorts. **(D)** Receiver operating characteristic (ROC) curves evaluating the diagnostic performance of each biomarker. Area Under the Curve (AUC) values are provided for discriminating ALS (red line), AD (green line), and PD (blue line) from healthy controls. Data in (C) are presented as individual data points with violin plots depicting the data distribution. Statistical significance across multiple groups was determined using one-way ANOVA followed by Tukey’s post hoc test (or the Kruskal-Wallis test followed by Dunn's multiple comparisons test for non-normally distributed data). *P < 0.05, P < 0.01, ***P < 0.001, ****P < 0.0001; ns = not significant.

**Figure S3. Plasma PF4 dynamics, BBB penetrance of systemic PF4, and its robust anti-inflammatory efficacy in the central nervous system.** **(A)** Time-course analysis of normalized plasma PF4 levels in wild-type (WT) and hSOD1^G93A^ mice across different disease progression stages (Day 70, 90, and 110). **(B)** Schematic representation of the in vivo biodistribution and targeted ELISA workflow. hSOD1^G93A^ mice received exogenous PF4 administration via either intravenous (i.v.) or intraperitoneal (i.p.) routes. To definitively exclude confounding signals from circulating blood, mice were subjected to extensive transcardial perfusion prior to the extraction of plasma, liver, spinal cord, and brain tissues. **(C and D)** Relative quantification of PF4 levels in peripheral and central tissues following i.v. (C) and i.p. (D) administration. Compared to vehicle-treated controls, both systemic delivery routes resulted in highly significant elevations of PF4 in the brain and spinal cord parenchyma, unequivocally demonstrating its capacity to cross the blood-brain barrier (BBB). **(E)** Absolute protein-level profiling of core pro-inflammatory mediators (TNF-α, C1q, CD11b, IL-1β, and NF-κB) in spinal cord homogenates. Systemic i.v. administration of PF4 dramatically blunted the severe neuroinflammatory cascade intrinsic to the hSOD1^G93A^ vehicle group, restoring marker expressions closer to WT physiological baselines. Data are presented as mean ± SEM (n = 5 mice per group). Statistical significance was determined using two-way ANOVA followed by Sidak's multiple comparisons test for time-course dynamics (A), an unpaired two-tailed Student's t-test for biodistribution comparisons (C, D), and one-way ANOVA followed by Tukey's post hoc test for multi-group inflammatory profiling (E). P < 0.01, ***P < 0.001, ****P < 0.0001.

**Figure S4. PF4 administration facilitates hSOD1 clearance and ameliorates disease progression in hSOD1^G93A^ mice.** **(A, B)** Representative Western blot (A) and quantitative densitometry (B) demonstrating the dose-dependent reduction of hSOD1 protein levels following PF4 treatment (0, 2.5, 5, and 10 ng/ml) in mouse spinal cord tissues (n = 4 independent biological replicates). **(C, D)** Western blot analysis (C) and corresponding quantification (D) of hSOD1 accumulation in the spinal cord of hSOD1^G93A^ mice. PF4 administration was initiated at either post-natal day 60 (P60, presymptomatic) or day 90 (P90, symptomatic) to evaluate intervention efficacy (n = 4 mice per group). **(E)** Longitudinal assessment of body weight dynamics. The left and middle panels illustrate the absolute body weight and relative body weight change over time, respectively. The right panel (violin plot) quantifies the net body weight change at day 112 comparing vehicle- and PF4-treated cohorts. **(F)** Kaplan-Meier survival curves (left) and violin plot quantification of overall survival duration (right) for WT, vehicle-treated, and PF4-treated hSOD1^G93A^ mice (n = 6 mice per group). **(G-J)** Comprehensive longitudinal evaluation of motor function and neurological decline, assessed via grip strength test (G), wirehang grip test (H), neurological severity score (I), and rotarod test (J) (n = 6 mice per group). Data are presented as mean ± SEM. Statistical significance was determined using one-way ANOVA followed by Dunnett's multiple comparisons test for (B), one-way ANOVA followed by Tukey's multiple comparisons test for (D), two-way ANOVA followed by Sidak's multiple comparisons test for time-course dynamics (E line graphs, G-J), Log-rank (Mantel-Cox) test for survival distribution (F, left), and an unpaired two-tailed Student's t-test for pairwise violin plot comparisons (E right, F right). P < 0.01, ***P < 0.001, ****P < 0.0001.

**Figure S5. Transcriptional analysis and pathway enrichment in SOD1-mutated iPSC-derived motor neurons.** **(A)** Volcano plot illustrating the differentially expressed genes (DEGs) in SOD1-mutated iPSC-derived motor neurons (iPSC-MNs) compared to healthy controls (Data source: GSE158264; n = 4 vs. n = 3 independent biological replicates). Significantly upregulated (red) and downregulated (blue) genes were defined by thresholds of |log2 fold change| > 0.58 and adjusted P-value < 0.05. **(B, C)** Heatmaps depicting the relative expression levels (Z-score normalized) of representative DEGs involved in the core macroautophagy machinery (B) and selective mitophagy/mitochondrial dynamics (C). **(D, F)** Gene Ontology (GO) biological process enrichment analysis of the DEGs. The top 20 significantly enriched pathways are visualized as a bubble plot (D, ranked by gene count and significance) and a corresponding bar chart (F, ranked by -log10 P-value). **(E)** Gene Set Enrichment Analysis (GSEA) plots evaluating specific functional pathways, including "regulation of autophagy", "negative regulation of autophagy", "mitochondrial DNA metabolic process", and "mitochondrial gene expression". Statistical significance for the transcriptomic analysis was determined using the empirical Bayes method in the **limma** R package, with GO and GSEA enrichment analyses performed via the **clusterProfiler** R package.

**Figure S6. Advanced functional enrichment visualization of DEGs in SOD1-mutated iPSC-motor neurons.** **(A)** Chord diagram detailing the precise interconnectivity between representative differentially expressed genes (DEGs, left hemisphere) and highly enriched Gene Ontology (GO) biological processes related to mitochondrial dynamics and autophagy (right hemisphere). **(B)** Circular GO enrichment plot summarizing the overarching functional landscape of the DEGs. The outer ring displays the significance level (-log10 P-value) of specific GO terms categorized into Biological Process (BP, red), Cellular Component (CC, purple), and Molecular Function (MF, blue). The inner tracks represent the total number of genes associated with each term and the proportional distribution of upregulated (pink) and downregulated (light blue) genes within those specific GO terms. (Data source: GSE158264).

**Figure S7. Assessment of basal autophagic flux in SOD1-expressing cells.** **(A, B)** Western blot analysis of autophagic flux. Representative blots (A) and corresponding densitometric quantification (B) of p62 and LC3 levels in EGFP control and SOD1 model cells treated with or without the lysosomal inhibitor chloroquine (CQ) (n = 4 independent biological replicates). **(C–E)** Immunofluorescence analysis of autophagic markers. Representative images showing the distribution of LC3 (C) and p62 (D) puncta co-stained with Cytochrome c (CYC) to visualize the mitochondrial network. Quantification of the mean fluorescence intensity per cell is shown in **(E)** (n = 20–30 cells per group from 6 independent biological replicates). Data are presented as mean ± SEM for bar graphs, and violin plots depict overall data distribution. Statistical significance was determined using one-way ANOVA followed by Tukey’s post hoc test. *P < 0.05, P < 0.01, ****P < 0.0001. Scale bars = 10 μm.

**Figure S8. Transcriptomic screening of potential upstream receptors for PF4.** Absolute expression levels, quantified as Transcripts Per Million (TPM), of established and putative candidate receptors (Lrp1, Gpc1, Gpc2, Itgb2, Sdc1, Sdc2, Sdc3, Sdc4, and Tlr4) in NSC-34 motor neuron-like cells expressing mutant SOD1 following vehicle or PF4 administration. The transcriptomic profiling reveals highly significant differential expression in specific receptor targets (notably Lrp1, Gpc1, Sdc1, and Tlr4), providing an unbiased, data-driven rationale for the targeted downstream mechanistic validation. Data are presented as mean ± SEM with individual data points representing independent biological replicates (n = 4 per group). Statistical significance was determined using two-way ANOVA followed by Sidak's multiple comparisons test. ***P < 0.001; ns, not significant.

**Figure S9. Original full-length Western blot images supporting the main text figures.** Molecular weight markers are indicated in kilodaltons (KDa) on the side of the blots. To ensure data integrity and experimental efficiency during Western blot analyses, membranes were physically cut prior to hybridization with primary antibodies based on the molecular weights of the target proteins to simultaneously probe multiple proteins and conserve reagents. Therefore, cropped edges and partial or single-sided molecular weight markers are visible in the representative full-length blots. Furthermore, where necessary, the full blots were digitally cropped to remove irrelevant experimental samples or parallel replicate lanes not directly related to the final presentation of this study.

**Figure S10. Original full-length Western blot images supporting the main text figures.** Molecular weight markers are indicated in kilodaltons (KDa) on the side of the blots. To ensure data integrity and experimental efficiency during Western blot analyses, membranes were physically cut prior to hybridization with primary antibodies based on the molecular weights of the target proteins to simultaneously probe multiple proteins and conserve reagents. Therefore, cropped edges and partial or single-sided molecular weight markers are visible in the representative full-length blots. Furthermore, where necessary, the full blots were digitally cropped to remove irrelevant experimental samples or parallel replicate lanes not directly related to the final presentation of this study.

**Figure S11. Original full-length Western blot images supporting the main text figures.** Molecular weight markers are indicated in kilodaltons (KDa) on the side of the blots. To ensure data integrity and experimental efficiency during Western blot analyses, membranes were physically cut prior to hybridization with primary antibodies based on the molecular weights of the target proteins to simultaneously probe multiple proteins and conserve reagents. Therefore, cropped edges and partial or single-sided molecular weight markers are visible in the representative full-length blots. Furthermore, where necessary, the full blots were digitally cropped to remove irrelevant experimental samples or parallel replicate lanes not directly related to the final presentation of this study.

**Figure S12. Single-color imaging controls confirm the absence of fluorescence bleed-through in confocal microscopy.** Representative 4×4 imaging matrix of single-color controls to experimentally rule out spectral crossover. Samples expressing or stained with only a single fluorophore—DAPI (excited at 405 nm), EGFP (excited at 488 nm), UBQLN2 (detected via a 561 nm-excitable fluorophore), or TOMM20 (detected via a 647 nm-excitable fluorophore)—were imaged across all four detection channels under exactly the same parameters used for multi-plexed imaging. The images unequivocally demonstrate that each specific fluorophore emits exclusively within its designated detection window (visible along the diagonal). Crucially, no emission signals are detected in any adjacent off-target channels (which remain completely black), thereby definitively validating the efficacy of the sequential scanning setup and eliminating the possibility of optical artifacts or fluorescence bleed-through in our co-localization analyses.

**Table S1. Key resources table**

| REAGENT or RESOURCE | SOURCE | IDENTIFIER | |  |
| --- | --- | --- | --- | --- |
| Antibody |  |  | |  |
| Rabbit anti-Cathepsin D (CTSD) | Proteintech | Cat# 21327-1-AP; RRID: AB_10733646 | |  |
| Rabbit anti-Cytochrome c (CYC) | Santa Cruz Biotechnology | Cat# sc-13156; RRID: AB_627385 | |  |
| Mouse anti-NeuN | Merck Millipore | Cat# MAB377; RRID: AB_2298772 | |  |
| Rabbit anti-SOD1 | Abcam | Cat# ab13498; RRID: AB_300402 | |  |
| Rabbit anti-TDP-43 | Proteintech | Cat# 12892-1-AP; RRID: AB_2200505 | |  |
| Rabbit anti-TAX1BP1 | Bioss | Cat# bsm-62170R; RRID: N/A | |  |
| Rabbit anti-NDP52 | Proteintech | Cat# 12229-1-AP; RRID: AB_11182600 | |  |
| Rabbit anti-ATG7 | Proteintech | Cat# 10088-2-AP; RRID: AB_2062351 | |  |
| Rabbit anti-HSP70 | Affinity Biosciences | Cat# AF5466; RRID: AB_2837950 | |  |
| Rabbit anti-CHOP | Affinity Biosciences | Cat# AF6277; RRID: AB_2835130 | |  |
| Rabbit anti-COX2 | Proteintech | Cat# 12375-1-AP; RRID: AB_2085127 | |  |
| Rabbit anti-PINK1 | ABclonal | Cat# A7131; RRID: AB_2767686 | |  |
| Rabbit anti-Parkin | ABclonal | Cat# A0968; RRID: AB_2757487 | |  |
| Rabbit anti-LC3B | Abcam | Cat# ab192890; RRID: AB_2827794 | |  |
| Rabbit anti-LRP1 | Proteintech | Cat# 26106-1-AP; RRID：AB_3085841 | |  |
| Rabbit anti-TBK1 | Proteintech | Cat# 83686-3-RR; RRID：AB_3671288 | |  |
| Rabbit anti-pTBK1 | CST | Cat# 5483; RRID:AB_10693472 | |  |
| Rabbit anti-OPTN (Optineurin) | Proteintech | Cat# 10837-1-AP; RRID: AB_2156665 | |  |
| Rabbit anti-pOPTN  (Optineurin) | CST | Cat# 31304; RRID:NA | |  |
| Rabbit anti-p62/SQSTM1 | Proteintech | Cat# 31403-1-AP; RRID: AB_3669966 | |  |
| Rabbit anti-UBQLN2 | ABclonal | Cat# A9568; RRID: AB_2772790 | |  |
| Mouse anti-GFAP | CST | Cat# 3670; RRID: AB_561049 | |  |
| Rabbit anti-Iba-1 | FUJIFILM Wako | Cat# 019-19741; RRID: AB_839504 | |  |
| Mouse anti-GFP | Proteintech | Cat# 66002-1-Ig; RRID: AB_11182611 | |  |
| Mouse anti-GAPDH | Solarbio | Cat# K200057M; RRID: AB_2943417 | |  |
| Mouse anti-β-Actin | Proteintech | Cat# 66009-1-Ig; RRID: AB_2687938 | |  |
| Mouse anti-SV2 | DSHB | RRID: AB_2315387 | |  |
| Mouse anti-SMI-32 | BioLegend | Cat# 801701; RRID: AB_2564642 | |  |
| Goat Anti-Rabbit IgG HRP | CST | Cat# 7074S; RRID: AB_2099233 | |  |
| Horse Anti-Mouse IgG HRP | CST | Cat# 7076S; RRID: AB_330924 | |  |
| Alexa Fluor 647 α-Bungarotoxin | Invitrogen | Cat# B35450; RRID: N/A | |  |
| Goat anti-Rabbit IgG (H+L), DyLight 549 | EarthOx | Cat# E032220; RRID: N/A | |  |
| Goat anti-Mouse IgG (H+L), DyLight 549 | EarthOx | Cat# E032310; RRID: N/A | |  |
| Goat anti-Rabbit IgG (H+L), DyLight 649 | EarthOx | Cat# E032620; RRID: N/A | |  |
| Goat anti-Mouse IgG (H+L), DyLight 649 | EarthOx | Cat# E032610; RRID: N/A | |  |
| Chemicals, Peptides, and Recombinant Proteins |  | |  | |
| DMEM (high glucose) | Gibco (Thermo Fisher) | | Cat# 11320033; | |
| Fetal Bovine Serum (FBS) | ZETA life | | Cat# Z7186FBS-500; | |
| Opti-MEM Reduced Serum Medium | Invitrogen (Thermo Fisher) | | Cat# 11668027; | |
| Lipofectamine 2000 Transfection Reagent | Invitrogen (Thermo Fisher) | | Cat# 31985062; | |
| G418 (Geneticin) | Sigma-Aldrich | | Cat# A1720 ; | |
| Chloroquine (CQ) | GLPBIO | | Cat# GC19549; | |
| CCCP | GLPBIO | | Cat# GC14727; | |
| PF4 (CXCL4), recombinant protein | ProSpec | | Cat# CHM-245; | |
| TMRE | Beyotime | | Cat# C2001S; | |
| MitoTracker Deep Red FM | GLPBIO | | Cat# GC20139; | |
| LysoTracker Red | GLPBIO | | Cat# GC19882; | |
| Hoechst 33342 | Biosharp | | Cat# BL803A; | |
| RIPA Lysis Buffer | Beyotime | | Cat# P0013B; | |
| Protease inhibitor cocktail | GLPBIO | | Cat# GK10014 | |
| Isopentane | Macklin | | Cat# I814047; | |
| Cresyl Violet | Sigma-Aldrich | | Cat# C5042; | |
| GA-RNA Transfection Reagent | GENEADV | | Cat# GAT1001; | |
| ELISA kits |  | |  | |
| Platelet factor 4 (PF4 / CXCL4) | R&D Systems (Bio-Techne) | | Cat# DPF40 | |
| Mouse PF4 ELISA Kit (CXCL4) | Abcam | | Cat# ab100735 | |
| Brain‑derived neurotrophic factor (BDNF) | Abcam | | Cat# ab212166 | |
| Vascular endothelial growth factor A (VEGF, human) | Abcam | | Cat# ab222510 | |
| Mouse TNF alpha ELISA Kit | Abcam | | Cat# ab208348 | |
| Mouse C1q ELISA Kit | Abcam | | Cat# ab291069 | |
| Mouse IL-1 beta/IL-1F2 Quantikine ELISA Kit | R&D Systems | | Cat# MLB00C | |
| NF kappaB p65 (pS536) ELISA Kit | Abcam | | Cat# ab176647 | |
| Mouse Integrin alpha-M (Itgam/CD11b) ELISA Kit | Cusabio | | Cat# CSB-E17417m | |

| Critical Commercial Assays |  |  |
| --- | --- | --- |
| Cell Counting Kit-8 (CCK-8) | GLPBIO | Cat# GK10001; |
| BCA Protein Assay Kit | GLPBIO | Cat# GK10009; |
| H&E Staining Kit | Solarbio | Cat# G1121; |

| Deposited Data |  |  |
| --- | --- | --- |
| UK Biobank data | UK Biobank | [https://www.ukbiobank.ac.uk](https://www.ukbiobank.ac.uk/) |
| GEO dataset: GSE158264 | NCBI GEO | GSE158264 |
| Experimental Models: Cell Lines |  |  |
| NSC-34 | — | RRID: CVCL_D356 |
| HEK293 | — | RRID: CVCL_0045 |
| Experimental Models: Organisms/Strains |  |  |
| Mouse: B6SJL-Tg(SOD1*G93A)1Gur/J | The Jackson Laboratory | Strain # 002726; RRID: IMSR_JAX:002726 |
| Mouse: C57BL/6J | The Jackson Laboratory | Strain #:000664; RRID: IMSR_JAX:000664 |
| Oligonucleotides |  |  |
| siRNA targeting ATG7 | GENEADV | Cat# PC00282700 |
| siRNA targeting OPTN | GENEADV | Cat# PC00282689 |
| siRNA targeting UBQLN2 | GENEADV | Cat# PC00282687 |
| siRNA targeting PINK1 | GENEADV | Cat# PC00282688 |
| siRNA targeting LRP1 | GenePharma | N/A |
| Recombinant DNA |  |  |
| pTRE3G-mCherry-BI-EGFP | Gift (Hebei Medical University) | N/A |
| pTRE3G-mCherry-BI-EGFP-SOD1^G93A^ | Gift (Hebei Medical University) | N/A |
| pTRE3G-mCherry-BI-EGFP-TDP-25-PrLD | Gift (Hebei Medical University) | N/A |
| pCMV-EGFP-(GA)_30_ | This paper | N/A |
| pCMV-EGFP-(GR)_30_ | This paper | N/A |
| pEX-3-mCherry-EGFP-LC3 | GenePharma | N/A |
| Oligonucleotides |  |  |
| Primer: hSOD1-F | This paper | Sequence: 5'-CATCAGCCCTAATCCATCTGA-3' |
| Primer: hSOD1-R | This paper | Sequence: 5'-CGCGACTAACAATCAAAGTGA-3' |
| Software and Algorithms |  |  |
| R (v4.4.1) | R Core Team | [https://www.r-project.org](https://www.r-project.org/) |
| GraphPad Prism (v9.5.0) | GraphPad Software | RRID: SCR_002798 |
| Fiji (ImageJ) | NIH | RRID: SCR_002285 |
| Image Lab (v6.1) | Bio-Rad | RRID: SCR_014210 |
| Majorbio Cloud Platform | Majorbio | [www.majorbio.com](http://www.majorbio.com/) |
| Other Equipment |  |  |
| Rotarod apparatus | Xinruan | XR1514 |
| Grip Strength Meter | Xinruan | XR-YLS-13A |
| High-resolution laser confocal microscope | Leica | STELLARIS 5 |
| Cryostat | Leica | CM1950 |
| Varioskan Flash Multimode Reader | Thermo Fisher | Cat# 5250510 |
| Transmission electron microscopy | JEOL | JEM-F200 |

**Table S2. Demographic characteristics and biomarker levels stratified by age**

|  | Age < 30 | Age 30-65 | | | Age > 65 | | |
| --- | --- | --- | --- | --- | --- | --- | --- |
| Characteristic | Control **(n=20)** | Control  **(n=20)** | ALS  **(n=20)** | P-value  **(n=20)** | Control  **(n=20)** | ALS  **(n=20)** | P_value |
| Sex |  |  |  | 0.752 |  |  | 0.301 |
| Female | 8 (40.0%) | 11 (55.0%) | 9 (45.0%) |  | 4 (20.0%) | 8 (40.0%) |  |
| Male | 12 (60.0%) | 9 (45.0%) | 11 (55.0%) |  | 16(80.0%) | 12(60.0%) |  |
| PF4 (μg/ml) |  | / |  | 0.317 |  |  | **0.008**** |
| Mean (SD) | 14 (7.3) | 10 (4.8) | 9.0 (2.2) |  | 7.2 (2.8) | 5.1 (1.8) |  |
| BDNF(ng/ml) |  |  |  | 0.0926 |  |  | 0.123 |
| Mean (SD) | 24 (8.9) | 22 (1.8) | 20 (5.5) |  | 23 (2.2) | 19 (11) |  |
| VEGF (pg/ml) |  |  |  | 0.646 |  |  | 0.682 |
| Mean (SD) | 110 (39) | 130 (22) | 140 (120) |  | 140 (29) | 150 (82) |  |

***p < 0.05**

**TableS3. Demographic characteristics and multiplex biomarker profiles of the expanded cross-sectional cohort**

| **Characteristic** | **HC (n=60)** | **ALS (n=60)** | **AD (n=60)** | **PD (n=60)** | **P-value** |
| --- | --- | --- | --- | --- | --- |
| **Age (years)** |  |  |  |  | <0.001*** |
| Mean (SD) | 58.3 (6.5) | 58.7 (6.9) | 62.5 (5.8) | 61.1 (5.5) |  |
| **Sex** |  |  |  |  | 0.171 |
| Female | 23 (38.3%) | 24 (40.0%) | 33 (55.0%) | 31 (51.7%) |  |
| Male | 37 (61.7%) | 36 (60.0%) | 27 (45.0%) | 29 (48.3%) |  |
| **PF4 (ng/mL)** |  |  |  |  | 0.012* |
| Mean (SD) | 1158.7 (287.5) | 994.0 (374.2) | 1143.7 (283.3) | 1114.7 (243.0) |  |
| **BDNF (ng/mL)** |  |  |  |  | <0.001*** |
| Mean (SD) | 25.2 (6.6) | 21.9 (8.0) | 19.1 (6.9) | 21.2 (8.5) |  |
| **VEGF (pg/mL)** |  |  |  |  | 0.498 |
| Mean (SD) | 152.2 (47.8) | 141.8 (44.1) | 139.2 (52.5) | 142.8 (53.0) |  |
| **PDGF_BB (pg/mL)** |  |  |  |  | 0.684 |
| Mean (SD) | 15.7 (5.0) | 15.9 (5.2) | 15.0 (5.3) | 15.0 (4.7) |  |
| **CCL5 (ng/mL)** |  |  |  |  | <0.001*** |
| Mean (SD) | 42.3 (12.4) | 55.2 (16.2) | 51.7 (14.8) | 48.0 (15.2) |  |
| **sP_selectin (ng/mL)** |  |  |  |  | 0.037* |
| Mean (SD) | 31.3 (12.2) | 37.1 (14.2) | 36.6 (12.2) | 33.6 (10.8) |  |
| **IGF_1 (ng/mL)** |  |  |  |  | 0.230 |
| Mean (SD) | 109.1 (26.6) | 101.5 (27.7) | 109.0 (26.2) | 102.3 (26.4) |  |
| **Serotonin (ng/mL)** |  |  |  |  | <0.001*** |
| Mean (SD) | 125.3 (50.8) | 90.2 (47.7) | 113.7 (41.8) | 110.7 (36.7) |  |
| **TGF_b1 (ng/mL)** |  |  |  |  | 0.001** |
| Mean (SD) | 20.5 (7.2) | 26.1 (8.6) | 23.5 (8.1) | 25.1 (8.3) |  |
| **CXCL7 (ng/mL)** |  |  |  |  | 0.332 |
| Mean (SD) | 799.5 (228.3) | 769.3 (287.2) | 851.6 (239.5) | 810.9 (226.2) |  |

*p < 0.05, **p < 0.01, ***p < 0.001. P-values were calculated using ANOVA for continuous variables and Chi-square test for categorical variables.

**Table S4. ROC analysis results of Platelet-related factors for ALS vs HC**

| **Biomarkers** | **AUC** | **95% CI** | **Cut-off Value** | **Sensitivity** | **Specificity** |
| --- | --- | --- | --- | --- | --- |
| PF4 | 0.769 | 0.702 - 0.837 | -0.38 | 63.0% | 85.0% |
| BDNF | 0.605 | 0.527 - 0.684 | -0.40 | 48.0% | 75.0% |
| VEGF | 0.568 | 0.489 - 0.648 | -0.17 | 51.0% | 66.0% |
| PF4+BDNF+VEGF | 0.778 | 0.712 - 0.844 | 0.54 | 65.0% | 82.0% |
| **ALS: Amyotrophic Lateral Sclerosis; HC: Healthy Control; 95% CI: 95% Confidence Interval.** | | | | | |
